# Autonomous metal-organic framework nanorobots for active mitochondria-targeted cancer therapy

**DOI:** 10.1126/sciadv.adh1736

**Published:** 2023-06-09

**Authors:** Xiqi Peng, Songsong Tang, Daitian Tang, Dewang Zhou, Yangyang Li, Qiwei Chen, Fangchen Wan, Heather Lukas, Hong Han, Xueji Zhang, Wei Gao, Song Wu

**Affiliations:** ^1^ Luohu Clinical Institute of Shantou University Medical College, Shantou University Medical College, Shantou 515000, P. R. China.; ^2^ Institute of Urology, The Third Affiliated Hospital of Shenzhen University, Shenzhen 518000, P. R. China.; ^3^ Andrew and Peggy Cherng Department of Medical Engineering, California Institute of Technology, Pasadena, CA 91125, USA.; ^4^ School of Biomedical Engineering, Health Science Centre, Shenzhen University, Shenzhen 518060, P. R. China.; ^5^ Department of Urology, South China Hospital, Medical School, Shenzhen University, Shenzhen 518116, P. R. China.

## Abstract

Nanorobotic manipulation to access subcellular organelles remains unmet due to the challenge in achieving intracellular controlled propulsion. Intracellular organelles, such as mitochondria, are an emerging therapeutic target with selective targeting and curative efficacy. We report an autonomous nanorobot capable of active mitochondria-targeted drug delivery, prepared by facilely encapsulating mitochondriotropic doxorubicin-triphenylphosphonium (DOX-TPP) inside zeolitic imidazolate framework-67 (ZIF-67) nanoparticles. The catalytic ZIF-67 body can decompose bioavailable hydrogen peroxide overexpressed inside tumor cells to generate effective intracellular mitochondriotropic movement in the presence of TPP cation. This nanorobot-enhanced targeted drug delivery induces mitochondria-mediated apoptosis and mitochondrial dysregulation to improve the in vitro anticancer effect and suppression of cancer cell metastasis, further verified by in vivo evaluations in the subcutaneous tumor model and orthotopic breast tumor model. This nanorobot unlocks a fresh field of nanorobot operation with intracellular organelle access, thereby introducing the next generation of robotic medical devices with organelle-level resolution for precision therapy.

## Teaser

Autonomous MOF-based nanorobots enable active and targeted drug delivery to intracellular organelles for cancer therapy.

## INTRODUCTION

Micro-/nanorobots have offered remarkable revolutions for biomedical applications benefiting from their autonomous movement (*1*, *2*). These tiny machines can harness local energy [e.g., magnetic (*3*, *4*), chemical (*5*, *6*), acoustic (*7*–*9*), and light (*10*, *11*)] to generate propulsive force, enabling effective movement within confined spaces and successful transport to hard-to-reach sites, such as blood vessels (*12*), the vitreous (*13*), and the lungs (*14*). Given that cellular dysfunction directly alters the homeostasis of living organisms to give rise to diverse diseases, recent efforts have extended the operation scope of medical robots from the organ level down to the cellular level, allowing precise operations to therapeutically regulate cellular dynamics. Pioneer works have reported various intracellular applications of nanorobots, such as motion within cells (*15*, *16*), rapid internalization for intracellular delivery [e.g., small interfering RNA (*17*), oxygen (*18*), enzyme (*19*)], intracellular sensing (*20*, *21*), and scavenging of reactive oxygen species (ROS) (*22*). In addition, nanorobots may help regulate cellular metabolism by targeting the subsystem of organelles involved, such as the nucleus, lysosome, mitochondrion, endoplasmic reticulum, and Golgi apparatus (*23*). The activity and chemical composition of these subcellular organelles alters cell metabolism, directly determining the homeostasis of living systems (*24*). Targeting these organelles shows great therapeutic potential to enhance drug delivery and treatment efficacy of prevalent pathologies (*25*–*27*). However, the subcellular manipulation of nanorobots to access the specific organelles within the cytoplasm remains a bottleneck. The lack of directed mobility and manipulation within the cell has limited the development and translation of nanorobots for cellular modulation.

**Fig. 1.**
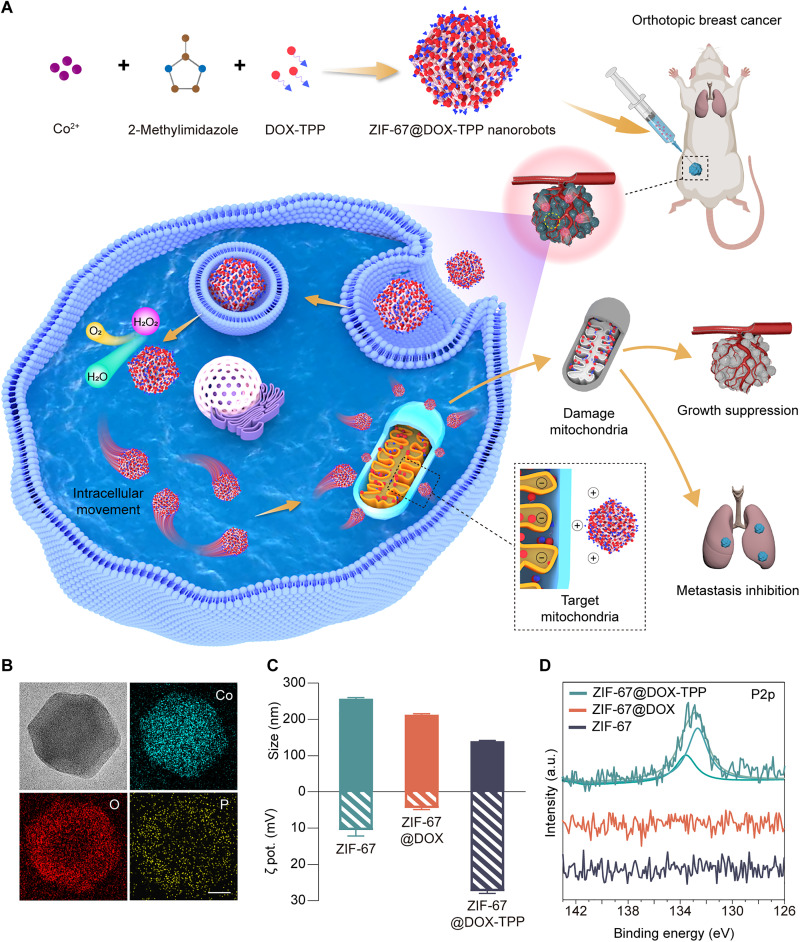
Overall concept of active mitochondria-targeted cancer therapy and characterization of ZIF-67@DOX-TPP nanorobots. (**A**) Schematic of the fabrication of ZIF-67@DOX-TPP nanorobots and their intracellular mitochondriotropic propulsion, enabling mitochondrial targeting drug delivery to effectively inhibit cancer growth and metastasis. (**B**) Transmission electron microscopy (TEM) and energy-dispersive x-ray images of ZIF-67@DOX-TPP nanorobots. Scale bars, 50 nm. (**C**) Size and zeta (ζ) potential (*n* = 3; means ± SD) and (**D**) P2p x-ray photoelectron spectroscopy (XPS) spectra of zeolitic imidazolate framework-67 (ZIF-67) nanoparticles (NPs), ZIF-67@DOX, and ZIF-67@DOX-TPP nanorobots. DOX, doxorubicin; TPP, triphenylphosphonium; a.u., arbitrary units.

Herein, we present a self-powered metal-organic framework (MOF)–based nanorobot capable of active and targeted drug delivery to mitochondria for cancer eradication and metastasis inhibition (Fig. 1A). Mitochondria are used as the therapeutic target due to their pivotal role in adenosine triphosphate (ATP) production, calcium regulation, cellular metabolism, and apoptosis in eukaryotic cells (*28*). Mitochondrial dysfunction has been demonstrated to contribute to various common pathologies, such as cancer growth and metastasis, inflammation, and neurodegeneration (*29*). Zeolitic imidazolate framework-67 (ZIF-67) capable of hydrogen peroxide (H_2_O_2_) catalysis was selected as the material foundation of the nanorobot, serving as the power engine (*30*). The chemotherapeutic drug, doxorubicin (DOX), conjugated with mitochondriotropic triphenylphosphonium (TPP^+^) cation (denoted as DOX-TPP), was chosen to enhance the binding of nanorobots with mitochondria (*31*). The lipophilic TPP cation leverages the high mitochondrial membrane potential to passively target the mitochondria (*26*, *32*).

In this work, the self-propelled nanorobots were facilely fabricated by encapsulating DOX-TPP inside ZIF-67 nanoparticles (NPs) (denoted as ZIF-67@DOX-TPP) (Fig. 1A). The catalytic decomposition of ZIF-67 in the presence of H_2_O_2_, which is overproduced inside tumor cells, generates sufficient force to propel internalized ZIF-67@DOX-TPP nanorobots in the cytosol. Meanwhile, the encapsulated mitochondriotropic TPP^+^ leads to mitochondria-targeted movement of untethered nanorobots, yielding a targeted accumulation of nanorobots and subsequently higher local drug concentration at the mitochondria. The controlled access to the mitochondria allows the regulation of mitochondrial dynamics for cancer therapy benefiting from the key role of mitochondria in modulating cancer growth and metastasis. The mitochondria-targeted propulsion of nanorobots could induce mitochondria-mediated apoptosis and mitochondrial dysfunction, leading to improved anticancer effects against various types of cancer cells and inhibited cancer cell migration and invasion for in vitro tests. Two animal models, the subcutaneous tumor model and the lung metastasis model (orthotopic breast cancer model), were used to validate the enhanced antitumor efficacy and suppression of cancer metastasis by ZIF-67@DOX-TPP nanorobots. Overall, this design expands the field of nanorobot operation to the organelle level with nanorobots acting as miniaturized intracellular surgeons to directly modulate cellular physiology for the treatment of pathological disorders. Developing medical robotic tools with organelle-targeted capabilities presents an advanced generation for precision therapy to achieve maximized therapeutic outcomes with reduced drug dosage.

## RESULTS

### Preparation and characterization of ZIF-67@DOX-TPP nanorobots

ZIF-67@DOX-TPP nanorobots were facilely fabricated via an in situ biomineralization approach by mixing DOX-TPP with the precursors of ZIF-67, cobalt nitrate, and 2-methylimidazole (*33*). ZIF-67 and DOX-encapsulating ZIF-67 (ZIF-67@DOX) NPs were also synthesized as counterparts. The transmission electron microscopy (TEM) images of both ZIF-67@DOX-TPP nanorobots (Fig. 1B and fig. S1, C and D) and ZIF-67 NPs (fig. S1, A and B) illustrated uniform size distribution with the typical morphology of rhombic dodecahedron (*34*), revealing the negligible impact of drug encapsulation on the structure of ZIF-67 NPs. The homogenous distribution of Co, O, and P in elemental mapping images of nanorobots (Fig. 1B) indicates the successful construction of the ZIF-67 entity, DOX, and TPP molecules, respectively. The nanorobots were examined with an average size of 140.0 nm and a zeta potential of 27.3 mV (Fig. 1C). The size of the nanorobots was smaller compared to ZIF-67 NPs, which might be attributed to the specific interaction between DOX-TPP and metal ions with enhanced coordination bonding (*35*, *36*). The nanorobot size (175.6 nm) in the TEM image (Fig. 1B) made up a high proportion in the hydrodynamic size distribution of nanorobots (fig. S2), indicating a consistency in size measurements across different methods. The x-ray photoelectron spectroscopy (XPS) analysis confirmed that the P element (fig. S3, A to D) and two distinct peaks in P 2p spectra (Fig. 1D) were only observed in ZIF-67@DOX-TPP nanorobots and were absent in ZIF-67 and ZIF-67@DOX NPs. The x-ray diffraction (XRD) analysis showed declined intensity of diffraction peaks of ZIF-67 after loading DOX and DOX-TPP due to the coordination between Co^2+^ and encapsulated compounds (fig. S3E) (*37*). The ultraviolet-visible (UV-vis) spectra of nanorobots illustrated broader absorptions involving the peaks of building blocks and encapsulated drugs (fig. S3F). The construction of ZIF-67@DOX-TPP nanorobots was further confirmed using fluorescent characterization by leveraging the fluorescence of DOX-TPP (fig. S4).

### Propulsion performance

ZIF-67, the active material of the nanorobot shell, can decompose H_2_O_2_ into water and oxygen due to the catalytic cobalt ion center (*30*). Geometric irregularities are inevitable during the fabrication of the rhombic dodecahedral ZIF-67, generating an asymmetric MOF structure. Thereby, the catalytic reaction of H_2_O_2_ around ZIF-67@DOX-TPP nanorobots induces uneven distribution of decomposed products with directional flow, propelling the nanorobot in self-diffusiophoretic movement (*38*–*40*).

**Fig. 2.**
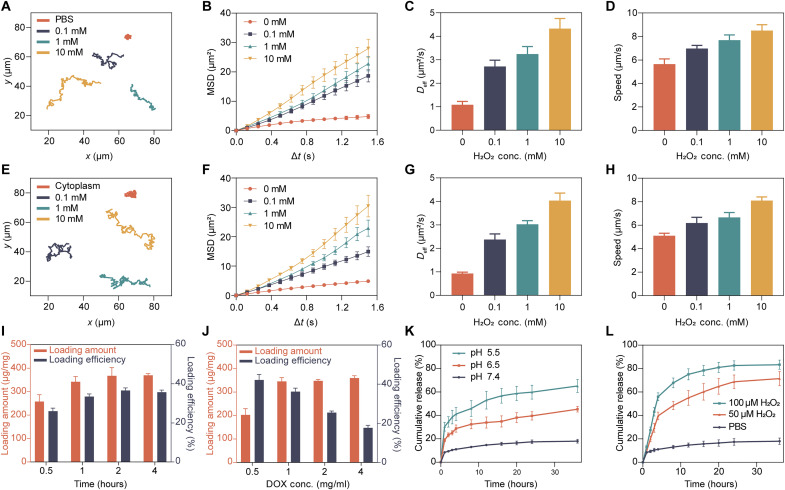
Motion study, drug loading, and drug release profiles of ZIF-67@DOX-TPP nanorobots. (**A**) Typical motion trajectories (over 20 s), (**B**) mean square displacement (MSD), (**C**) diffusion coefficient (*D*_eff_), and (**D**) speed of ZIF-67@DOX-TPP nanorobots in phosphate-buffered saline (PBS) solution with different H_2_O_2_ concentrations (*n* = 15; means ± SEM). (**E**) Typical movement trajectories (over 20 s), (**F**) MSD, (**G**) *D*_eff_, and (**H**) speed of ZIF-67@DOX-TPP nanorobots in the solution of cytoplasmic extracts with various H_2_O_2_ concentrations (*n* = 15; means ± SEM). Loading capacity of ZIF-67@DOX-TPP nanorobots upon various incubation time (**I**) and DOX-TPP input concentrations (**J**) (*n* = 3; means ± SD). The cumulative release of DOX-TPP from nanorobots upon different pH values (**K**) and H_2_O_2_ concentrations (**L**) (*n* = 3; means ± SEM).

The propulsion performance of ZIF-67@DOX-TPP nanorobots was first evaluated in phosphate-buffered saline (PBS) solution with various concentrations of H_2_O_2_ (0, 0.1, 1, and 10 mM). The nanorobots showed enhanced movement with elongated motion trajectories upon the rising concentration of H_2_O_2_ fuel (Fig. 2A and movie S1). The calculated mean square displacement (MSD) raised linearly with time and improved by the increasing H_2_O_2_ concentrations (Fig. 2B). The diffusion coefficient (*D*_eff_; Fig. 2C) and speed (Fig. 2D) of nanorobots also increased upon the raised H_2_O_2_ concentration. The catalytic propulsion of ZIF-67 NPs was also examined in 100 μM H_2_O_2_ and showed only slight difference compared to that of ZIF-67@DOX-TPP nanorobots (fig. S5 and movie S2), indicating the negligible influence of drug loading on the catalytic performance of ZIF-67. To expand the realm of operation of ZIF-67@DOX-TPP nanorobots to various biological environments, we explored their motion behavior in 1640 cell culture medium (fig. S6 and movie S3) and extracted cytoplasm (Fig. 2, E to H, and movie S4). The movement of nanorobots was enhanced by increasing H_2_O_2_ concentration from 0 to 10 mM in both media, where the motion parameters of nanorobots were comparable with that in PBS solution. Overall, these results demonstrate that ZIF-67@DOX-TPP nanorobots present effective propulsion in various biological fluids with H_2_O_2_ fuel, including cytoplasmic extraction, which is a prerequisite for their subsequent intracellular applications.

### Drug loading and release

Next, we explored the loading capacity and release behaviors of DOX-TPP in ZIF-67@DOX-TPP nanorobots. The loading capacity was explored upon various fabrication durations and drug inputs. At the DOX-TPP input of 1 mg/ml, the loading amount of DOX-TPP first increased and then saturated upon the increasing reaction time (Fig. 2I). The maximum loading amount (347.8 μg/mg) and efficiency (36.2%) were optimized at 2 hours of incubation. Increasing the concentration of DOX-TPP resulted in an increased loading amount of DOX-TPP until saturation, at which point excess DOX-TPP input resulted in decreased loading efficiency (Fig. 2J). Maximal loading capacity of DOX-TPP was optimally achieved at an input concentration of 1 mg/ml.

The DOX-TPP released from ZIF-67@DOX-TPP nanorobots was then analyzed in PBS solution under various pH and H_2_O_2_ conditions over 36 hours. Accelerated drug release was observed at the acidic pH of 5.5 (Fig. 2K) and higher H_2_O_2_ concentration (Fig. 2L). The nanorobot degradation was also examined to be accelerated in the solution with lower pH and higher H_2_O_2_ concentration using TEM characterization (fig. S7), contributing to the enhanced drug release behavior of nanorobots. The slow degradation of ZIF-67 body in neutral PBS was attributed to the binding affinity of phosphate species with metal centers, altering the coordination equilibrium of Co^2+^ and the organic ligand (*41*). Such degradation was accelerated in acidic conditions due to the intensified competition between proton and metal ion to coordinate with the organic linker (*42*) and in the presence of H_2_O_2_ due to the oxidizing breakage of the C═C and C═N bonds in the network (*43*). The increased fluid flow in the porous ZIF-67 structure during the propulsion of nanorobots in H_2_O_2_ solution may also improve the drug release of nanorobots. These release behaviors may promote the drug delivery of ZIF-67@DOX-TPP nanorobots in the tumor microenvironment with the acidic condition and high content of H_2_O_2_. The loading and release behaviors of ZIF-67@DOX were also evaluated (fig. S8) and were similar to that of ZIF-67@DOX-TPP nanorobots.

### Intracellular movement

**Fig. 3.**
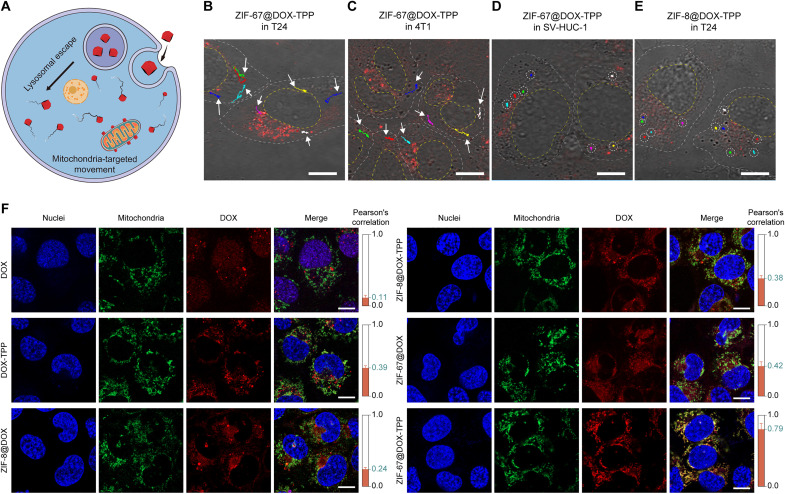
Intracellular autonomous propulsion and mitochondrial targeting of ZIF-67@DOX-TPP nanorobots. (**A**) Schematic of the mitochondria-targeted movement of nanorobots inside the tumor cell. Confocal laser scanning microscopy (CLSM) images of the merged optical and DOX channels showing intracellular motion trajectories of ZIF-67@DOX-TPP nanorobots inside the T24 bladder tumor cell (**B**), 4T1 breast tumor cell (**C**), and the SV- HUC-1 human uroepithelial cell (**D**). DOX fluorescence (red) represents ZIF-67@DOX-TPP nanorobots. Scale bars, 10 μm. (**E**) CLSM image showing intracellular motion trajectories of ZIF-8@DOX-TPP NPs inside the T24 cell. Scale bar, 10 μm. (**F**) Representative fluorescence images showing mitochondrial colocalization in T24 cells upon various incubations for 12 hours, including DOX, DOX-TPP, ZIF-8@DOX, ZIF-8@DOX-TPP, ZIF-67@DOX, and ZIF-67@DOX-TPP nanorobots. Nuclei were stained by Hoechst 33342 (blue). Mitochondria were labeled with MitoTracker Green FM (green). The red fluorescence represents the loaded DOX or DOX-TPP. The right columns show the calculated Pearson's correlation coefficients of the colocalization of mitochondria and nanorobots in T24 cells (*n* = 5; means ± SD). Scale bars, 10 μm.

The overexpressed H_2_O_2_ in cancer cells (up to 100 μM) offers bioavailable fuel to propel ZIF-67–based nanorobots (Fig. 3A) (*44*–*46*). The biocompatible endocytosis of the nanorobots by tumor cells and subsequent lysosome release are keys to the nanorobots' transport into the cytoplasm and their intracellular operation (*47*). ZIF-67 NPs were first examined with good biocompatibility with negligible toxicity to various cell lines below the concentration of 128 μg/ml (fig. S9), which was the operation range in our study. Regarding lysosome escape, ZIF-67@DOX-TPP nanorobots were incubated with T24 bladder tumor cells for 8 hours. The cellular nuclei and lysosomes were then stained with Hoechst 33342 and LysoTracker Green dye, respectively. The colocalization ratio of the lysosome and DOX fluorescence was calculated through the Pearson's correlation coefficient (PCC; the right columns in fig. S10) (*32*, *48*). The nanorobots spread to the cytosol as demonstrated by the intracellular dispersion of DOX fluorescence (red) and decreased PCC value from 0.67 to 0.35 over the incubation period (fig. S10). These results suggest successful cellular uptake, lysosomal release, and cytosolic dispersion of the nanorobots.

Confocal laser scanning microscopy (CLSM) was used to examine the intracellular motion of ZIF-67@DOX-TPP nanorobots. The fluorescence channel of CLSM was used to enhance the contrast of nanorobots inside the cell, enabling the visualization of DOX fluorescence (red) of the nanorobots along with the observation of bright field. ZIF-67@DOX-TPP nanorobots exhibited effective motion tracking with colored trajectories inside the T24 bladder cancer cell (Fig. 3B and movie S5) and 4T1 breast cancer cell (Fig. 3C and movie S5). When switching the tumor cell to a human uroepithelial cell line (SV-HUC-1), only Brownian motion and negligible displacement of nanorobots were observed in the normal cell due to the ultralow intracellular H_2_O_2_ level (Fig. 3D and movie S5) (*46*, *49*). To further confirm the essential role of ZIF-67 in such motion behavior, another type of MOF, ZIF-8, lacking the catalytic property to H_2_O_2_, was used to encapsulate DOX-TPP (ZIF-8@DOX-TPP) (*33*). ZIF-8@DOX-TPP NPs with the rhombic dodecahedral morphology also showed smaller size (166.0 nm) compared to ZIF-8 NPs (195.8 nm) (fig. S11, A and B), which is similar to the size difference between ZIF-67@DOX-TPP nanorobots and ZIF-67 NPs. As expected, the absence of the catalytic power only led to Brownian motion of ZIF-8@DOX-TPP in PBS at various H_2_O_2_ concentrations (fig. S11, C to F, and movie S6). The Brownian motion was slightly enhanced by increasing H_2_O_2_ concentration, which may be attributed to the lower kinematic viscosity and enhanced degradation with smaller particles in higher H_2_O_2_ concentration (*50*). ZIF-8 NPs exhibited similar motion behavior compared to ZIF-8@DOX-TPP NPs in 100 μM H_2_O_2_ (fig. S11, G and H, and movie S7). The Brownian motion of ZIF-8@DOX-TPP NPs was also observed within the T24 tumor cell (Fig. 3E and movie S5). These results demonstrate the effective propulsion of ZIF-67@DOX-TPP nanorobots inside tumor cells, ascribed to the catalytic decomposition between ZIF-67 and overexpressed H_2_O_2_ fuel in cancerous cells.

### Active mitochondria-targeted behavior

The mitochondrial targeting capability was evaluated by incubating T24 cells with various solutions, including DOX, DOX-TPP, and ZIF-8 NPs that encapsulated with DOX (ZIF-8@DOX) or DOX-TPP (ZIF-8@DOX-TPP), ZIF-67@DOX, and ZIF-67@DOX-TPP nanorobots. The cell nuclei and mitochondria were labeled with Hoechst 33342 and MitoTracker Green dye, respectively, for fluorescent characterization (Fig. 3F). The colocalization ratio of the mitochondria and DOX fluorescence was calculated through the PCC, shown in the right columns of Fig. 3F. The PCC value of the DOX-TPP group (0.39) was higher than the DOX group (0.11). ZIF-8@DOX displayed low fluorescence colocalization percentage of mitochondria and DOX with a small PCC value of 0.24. Changing the cargo to DOX-TPP (ZIF-8@DOX-TPP) increased the PCC value to 0.38, affirming the preferential targeting of mitochondria by lipophilic and cationic TPP^+^ due to the high mitochondrial membrane potential (*31*). Comparable PCC was achieved in the groups of ZIF-67@DOX in contrast with that of the ZIF-8@DOX-TPP group, revealing that the chemical propulsion of ZIF-67–based NPs could enhance their adhesion with mitochondria possibly through increased collisions. The colocalization ratio was greatly enhanced by ZIF-67@DOX-TPP nanorobots with a much higher PCC value of 0.79. The PCC value was also shown to increase with extended incubation period of the nanorobots and T24 cells from 2 to 12 hours (fig. S12). The catalytic-driven movement thus works synergistically with loaded mitochondriotropic TPP^+^ to enhance the binding efficacy of ZIF-67@DOX-TPP nanorobots with mitochondria, yielding effective mitochondrial targeting behavior. Such active mitochondria-targeted performance of ZIF-67@DOX-TPP nanorobots was also validated in 4T1 breast cancer cell line (fig. S13), which exhibited similar trend as in T24 cells.

### In vitro antitumor and antimetastatic effect

**Fig. 4.**
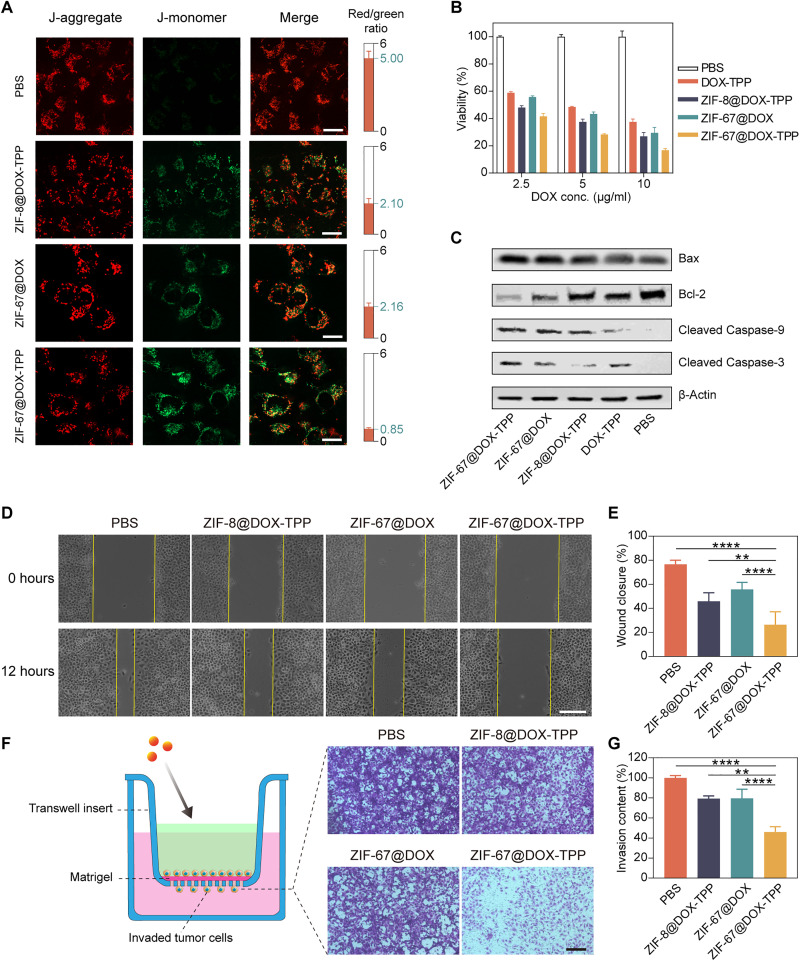
In vitro evaluation of ZIF-67@DOX-TPP nanorobots for cancer cell death and metastasis inhibition. (**A**) Fluorescence images of T24 cells stained with JC-1 dyes after incubated with various solutions for 8 hours, including PBS, ZIF-8@DOX-TPP, ZIF-67@DOX, and ZIF-67@DOX-TPP nanorobots. The right columns represent the calculated fluorescence ratio of J-aggregate (red) and J-monomer (green) (*n* = 5; means ± SD). Scale bars, 20 μm. (**B**) Viability of T24 cells after incubation with nanorobots and other control groups for 48 hours (*n* = 3; means ± SD). (**C**) Western blots for characteristic proteins involved in mitochondria-mediated apoptosis in T24 cells after treatment with nanorobots and other control groups. Bcl-2, B cell lymphoma 2. (**D**) Optical images showing in vitro wound healing assay and (**E**) corresponding wound closure percentages. The wound (cell gap) was built by a straight scratch across T24 cancer cells (0 hours). The wound closure rate was examined after incubation with nanorobots and other control groups for 12 hours (*n* = 5; means ± SD). Scale bar, 50 μm. (**F**) Schematic and images of invaded T24 cells across the Matrigel barrier after treatment with nanorobots and other control groups in the upper chamber of transwell assay and (**G**) corresponding invasive contents (*n* = 5; means ± SD). Scale bar, 100 μm. **P < 0.01; ****P < 0.0001; one-way analysis of variance (ANOVA).

The active mitochondrial targeting of ZIF-67@DOX-TPP nanorobots can enhance the drug accumulation around mitochondria, leading to improved mitochondrial damage and dysfunction. Because of the central role of mitochondria in controlling cell apoptosis and metastasis, such an effect on mitochondria is expected to induce mitochondria-mediated apoptosis and inhibited cell metastasis (*51*–*53*). This apoptotic pathway was first evaluated using JC-1 dye, a type of membrane-permeant probe for monitoring the mitochondrial membrane potential (*54*). The aggregated dye in healthy mitochondria fluoresces red, whereas, in damaged and depolarized mitochondria with low membrane potential, the monomeric dye fluoresces green (*55*). The ratio of red to green fluorescence can be used as an indicator of mitochondrial depolarization and early apoptosis. Here, T24 cells were incubated with nanorobots and other constructs for 8 hours and then stained with JC-1 dye. T24 cells that incubated with ZIF-67@DOX-TPP nanorobots exhibited the smallest red/green ratio of 0.85 compared to controls (Fig. 4A and fig. S14), suggesting the capability of nanorobots to evoke a stronger and earlier apoptotic response.

Then, we evaluated the viability of T24 cells that were cultured with nanorobots and other constructs for 48 hours. The DOX concentration in the drug-loaded constructs was tested at 2.5, 5, and 10 μg/ml. The highest cell-killing efficacy was achieved in the group of ZIF-67@DOX-TPP nanorobots in contrast with other constructs (Fig. 4B). To examine the influence of oxygen generated by H_2_O_2_ decomposition on the anticancer efficacy of DOX-TPP, the mixture of free ZIF-67 NPs and H_2_O_2_ was used to generate oxygen and incubate with DOX-TPP and T24 cells for 48 hours. No apparent alteration occurred on the cell viability when cells were incubated with the mixture of ZIF-67, H_2_O_2_ and DOX-TPP, and other control groups (fig. S15), suggesting the negligible influence of H_2_O_2_ decomposition on the anticancer effect of DOX-TPP. To further elucidate the apoptotic mechanism, a Western blot was performed to examine the involved protein expression. Mitochondrial outer membrane permeabilization is a signaling pathway that initiates cell death, which is balanced by the proapoptotic protein Bax and antiapoptotic protein B cell lymphoma 2 (Bcl-2). The mitochondria-mediated apoptotic pathway activates Bax but suppresses the expression of Bcl-2, leading to the increased permeability of the mitochondrial outer membrane. This allows the release of intermembrane space proteins (e.g., cytochrome c) to trigger the caspase cascade, inducing the cleavage of Caspase-9 and Caspase-3 (*56*). Treatment with ZIF-67@DOX-TPP nanorobots resulted in the largest up-regulation of Bax, cleaved caspase-9, and cleaved caspase-3 and down-regulation of Bcl-2 in comparison to other constructs (Fig. 4C). These results verify the capability of ZIF-67@DOX-TPP nanorobots in stimulating mitochondria-regulated apoptosis to achieve enhanced anticancer efficacy due to their effective mitochondria-targeted drug delivery. Besides T24 cells, ZIF-67@DOX-TPP also exhibited a strong anticancer response against other kinds of cancer cells, including 4T1 breast cancer cells and DOX-resistant BIU-87/ADR bladder cancer cells (fig. S16), revealing the merit of active mitochondriotropic delivery in killing a broad array of cancer cells, even cell lines that have previously exhibited chemotherapeutic drug resistance.

The mitochondrial metabolism also plays a critical role in regulating cancer metastasis. As the powerhouse of the cell, mitochondria produce ATP for cellular activities, including cell migration and invasion (*52*). ROS generated by the mitochondrial electron transport chain have been shown to promote cancer migration by activating the phosphatidylinositol 3-kinase pathway (*57*, *58*). In addition, mitochondrial DNA mutations and dynamic morphological changes of mitochondria are involved in the alteration of metastatic behavior (*59*, *60*). The pivotal role of mitochondria in cancer metastasis encourages us to explore the impact of nanorobots on the metastatic competence of cancer cells. The in vitro wound healing assay was first conducted. A gap acting as the “wound” was created by linearly scratching the confluent T24 cells. Then, T24 cells were incubated with nanorobots and other control groups for 12 hours to examine the wound closure percentage. ZIF-67@DOX-TPP nanorobots induced the smallest closure rate (26.3%) compared to other groups (Fig. 4, D and E), representing their significant suppression of cell migration. A transwell migration assay using a Matrigel barrier was further performed to evaluate cell invasion. T24 cells were injected in the upper layer and incubated with various solutions for 24 hours (Fig. 4F). The addition of ZIF-67@DOX-TPP nanorobots resulted in the lowest invasion rate of T24 cells that migrated through the barrier (Fig. 4G), demonstrating much stronger inhibition on cancer cell invasion. This enhanced suppression of nanorobots on cell migration and invasion was also verified in 4T1 cells (fig. S17). Together, these results highlight the improved tumor cell apoptosis and the suppressed tumor cell migration by mitochondrial targeting nanorobots, leading to an effective anticancer and antimetastatic chemotherapeutic paradigm.

### In vivo anticancer efficacy against a subcutaneous tumor model

**Fig. 5.**
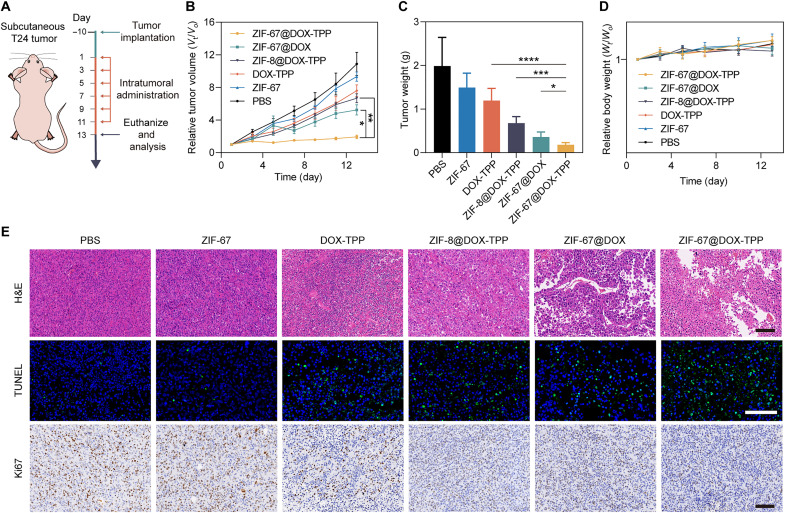
In vivo evaluation of the antitumor effect of ZIF-67@DOX-TPP nanorobots using a subcutaneous tumor model. (**A**) Schematic of the mouse model that bears subcutaneous T24 bladder tumor and the following treatment protocol. (**B**) The tumor growth kinetics of tumor-bearing mice that were treated with various intratumoral injections, including PBS, ZIF-67, DOX-TPP, ZIF-8@DOX-TPP, ZIF-67@DOX, and ZIF-67@DOX-TPP nanorobots, over the treatment process (*n* = 5; means ± SEM). (**C**) Excised tumor weights from mice at the end of treatment with nanorobots and other control groups (*n* = 5; means ± SD). (**D**) Body weight changes of tumor-bearing mice that were treated with nanorobots and other control groups over the treatment process (*n* = 5; means ± SEM). (**E**) Representative images of hematoxylin and eosin (H&E), terminal deoxynucleotidyl transferase–mediated deoxyuridine triphosphate nick end labeling (TUNEL), and Ki67 staining of resected tumor tissues from mice that were administrated with nanorobots and other control groups. Scale bars, 100 μm. *P < 0.05; **P < 0.01; ***P < 0.001; ****P < 0.0001; one-way ANOVA.

The in vivo antitumor effect of nanorobots was first evaluated using a subcutaneous T24 tumor model (Fig. 5A). The T24 tumor-bearing mice were intratumorally injected with ZIF-67@DOX-TPP nanorobots and other control groups. The tumor growth was monitored during the treatment course (Fig. 5B). The smallest tumor volume was achieved by the group of ZIF-67@DOX-TPP nanorobots, which was at least 2.71-fold lower than other groups. Such effective tumor inhibition of nanorobots was further verified by the weight of resected tumors after finishing the treatment (Fig. 5C and fig. S18). The excised tumors were also stained with hematoxylin and eosin (H&E), terminal deoxynucleotidyl transferase–mediated deoxyuridine triphosphate nick end labeling (TUNEL), and Ki67 (Fig. 5E). ZIF-67@DOX-TPP nanorobots induced more noticeable cell necrosis and apoptosis with inhibited proliferation rate compared to other conditions. The resected liver slices from the control and nanorobot groups and tumor slice from the nanorobot group were stained with HLA-DRA polyclonal antibody, which could label the HLA-DRA protein, one of the human leukocyte antigen (HLA) class II alpha chain paralogs and specifically expressed on human-derived cells. The tumor tissue showed the positive signal of HLA-DRA with brown color due to the interaction with human-derived T24 bladder cancer cells, whereas no signal (blue) was observed in the mouse liver slice (fig. S19). Such results indicate that the subcutaneous tumor did not develop any liver metastases during the treatment course.

Regarding the toxicity profile of nanorobots, no mice experienced distinct weight loss during the treatment course (Fig. 5D). Main organs of mice, including the heart, liver, spleen, lungs, and kidneys were processed with H&E staining (fig. S20). ZIF-67@DOX-TPP nanorobots had negligible influence on the tissue structure and integrity compared with the negative control (PBS), suggesting the favorable biosafety of ZIF-67@DOX-TPP nanorobots for in vivo applications.

### In vivo cancer treatment against orthotopic breast cancer

**Fig. 6.**
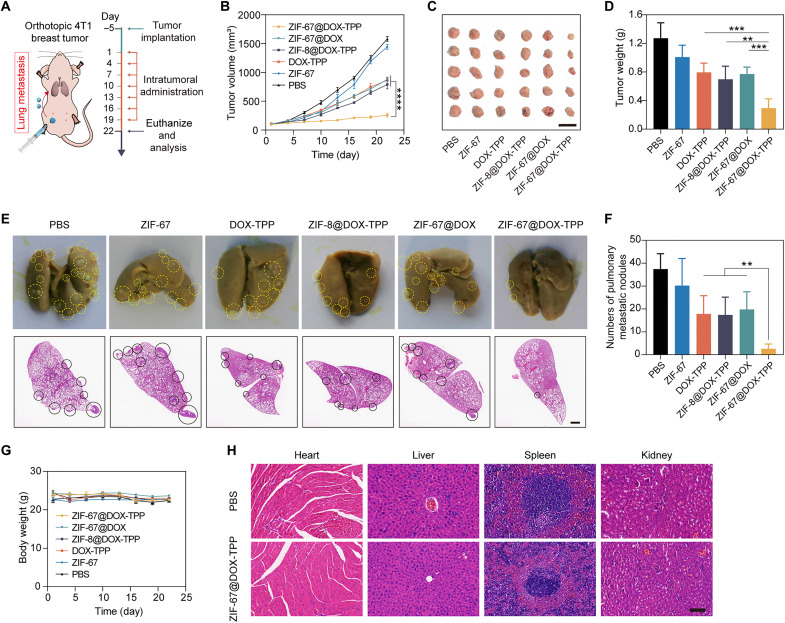
In vivo cancer treatment of ZIF-67@DOX-TPP nanorobots using an orthotopic breast tumor model. (**A**) Schematic of the mouse model bearing orthotopic 4T1 breast cancer and the following treatment protocol. (**B**) The tumor growth kinetics of tumor-bearing mice that were intratumorally injected with PBS, ZIF-67, DOX-TPP, ZIF-8@DOX-TPP, ZIF-67@DOX, and ZIF-67@DOX-TPP nanorobots over the treatment process (*n* = 5; means ± SD). (**C**) Tumor images and (**D**) weights of excised primary tumors from mice at the end of treatment with nanorobots and other control groups (*n* = 5; means ± SD). (**E**) Representative images and H&E staining showing metastatic nodules (labeled with circles) in resected lungs from tumor-bearing mice at the end of treatment with nanorobots and other control groups and (**F**) quantified pulmonary metastatic nodules (*n* = 5; means ± SD). Scale bar, 1 mm. (**G**) Body weight changes of tumor-bearing mice treated with nanorobots and other control groups over the treatment process (*n* = 5; means ± SD). (**H**) H&E staining of histological sections from main organs, including the heart, liver, spleen, and kidney, resected from tumor-bearing mice that were intratumorally injected with PBS or nanorobots at the end of the treatment course. Scale bar, 100 μm. **P < 0.01; ***P < 0.001; ****P < 0.0001; one-way ANOVA.

Next, we used an orthotopic unilateral breast tumor model to evaluate the dual function of ZIF-67@DOX-TPP nanorobots in inhibiting tumor growth and metastasis (Fig. 6A). The formed 4T1 tumors can spontaneously metastasize to distant organs, especially the lungs (*61*). The obtained tumor-bearing mice were treated with nanorobots or other control groups. The tumor growth and body weight of mice were monitored during the treatment course. After the treatment, the breast tumor was resected for imaging and weighting. Among the groups, ZIF-67@DOX-TPP nanorobots displayed the most effective inhibition of primary breast tumor growth with the smallest tumor volume (Fig. 6B) and weight (Fig. 6, C and D). To evaluate the pulmonary metastasis of breast tumor, the lungs of mice were dissected at the end point of the treatment course for imaging and H&E staining (Fig. 6E) to quantify the metastatic nodule (Fig. 6F). The average number of pulmonary metastatic nodules of ZIF-67@DOX-TPP nanorobots was quantified as 2.6, representing at least a sevenfold lower quantity than the groups of free DOX-TPP (17.8), ZIF-8@DOX-TPP (17.4), and ZIF-67@DOX (19.8). Verifying the biosafety of the nanorobots, no noticeable weight change of mice was observed (Fig. 6G). In addition, the results of H&E staining showed that the overall structure and integrity in the tissues of main organs were not affected by ZIF-67@DOX-TPP nanorobots and were comparable to the healthy control (PBS) (Fig. 6H). These results validate the function of ZIF-67@DOX-TPP nanorobots in effectively suppressing the growth and lung metastasis of orthotopic breast tumor model due to the active mitochondriotropic movement of nanorobots inside tumor cells.

## DISCUSSION

The capability of micro/nanorobots to move in a confined space at micro/nanoscale has brought distinct merits to cellular-level biomedical applications, such as intracellular delivery, sensing, and detoxification (*62*). However, major efforts have been devoted to cargo transport to the cell or cell vicinity. The interaction of nanorobots with subcellular organelles and their related impact on cellular dynamics have not yet been explored due to the barrier of achieving controlled propulsion within the restricted intracellular environment. On the other hand, organelles are the essential system to control cellular and body homeostasis. Treating organelles as the therapeutic target holds great potential to improve the targeting and therapeutic efficacy for drug delivery (*27*), but the development of organelle-targeted systems is impeded by passive nanoplatforms that lack the motility to reach desired sites.

To overcome these limitations, we have demonstrated MOF-based nanorobots with intracellular autonomous propulsion to actively target mitochondria inside tumor cells. ZIF-67–based nanorobots can catalyze the decomposition of the bioavailable H_2_O_2_ that is overproduced inside tumor cells to generate effective intracellular propulsion. The loaded mitochondriotropic TPP^+^ facilitates self-powered nanorobots to bind preferentially to mitochondria, overcoming the constraints of traditional passive nanosystems with limited transport efficacy to the specific organelle (*63*). This active targeting behavior leads to enhanced accumulation of nanorobots around mitochondria with higher local drug concentrations damaging and dysregulating the mitochondria. Compared to ZIF-67@DOX and static ZIF-8@DOX-TPP NPs, ZIF-67@DOX-TPP nanorobots led to improved anticancer efficacy against different types of cancer cells due to the induced mitochondria-mediated apoptosis. Such improved chemotherapeutic performance of the nanorobots was also verified in animal tests via the subcutaneous tumor model and the orthotopic breast tumor model. The capability to effectively kill different kinds of cancer cells demonstrates the promising potential to generalize this active targeted drug delivery to treat a wide range of cancerous sites. Moreover, the impact of ZIF-67@DOX-TPP nanorobots on mitochondria also greatly suppressed lung metastasis of orthotopic breast tumors.

Using bioavailable fuel to propel nanorobots with chemotactic guidance introduces a feasible route for the micro/nanorobotic community to create a robotic platform capable of effective and organelle-targeted propulsion inside cells (*1*), obviating the requirement for extra fuel and power sources. The catalytic MOF body, ZIF-67 showed enhanced degradation in the acidic and H_2_O_2_ conditions. Cancer cells are known to have dysregulated pH with a higher intracellular pH (>7.2) than the extracellular pH (~6.7 to 7.1) (*64*). The overexpression of intracellular H_2_O_2_ is another hallmark of tumor cells compared to normal cells (*44*, *45*). The nanorobot degradation in a practical intracellular environment thus would be mainly accelerated by the increased concentration of H_2_O_2_, with the potential for complete degradation within 1 week following the degradation trend shown in fig. S7. Such a biocompatible platform could fulfill the therapeutic mission without residual unwanted side effects and potentially meet rigorous safety requirements for clinical trial. Alternative catalytic MOFs with favorable biocompatibility and biodegradation could be readily used for future studies, such as Zr-based MOFs (e.g., UIO-66) and Fe-based MOFs (e.g., MIL-101) (*65*).

Overall, this work pioneers the subcellular manipulation of nanorobots with organelle-level resolution to modulate cellular functions, introducing a profound dimension for precision therapy with maximized therapeutic outcomes and reduced drug dosage. The active organelle-targeted system can be readily expanded to other types of catalytic entities (e.g., catalase), chemotactic molecules (e.g., dequalinium chloride), and organelles (e.g., nucleus, lysosome, and endoplasmic reticulum) toward the treatment of broader pathologies, such as neurodegenerative diseases (*66*) and atherosclerosis (*67*).

## MATERIALS AND METHODS

### Fabrication of ZIF-67@DOX-TPP nanorobots

A total of 9.7 mg of cobaltous nitrate hexahydrate [Co(NO_3_)_2_·6H_2_O; Aladdin] was first dissolved in 1 ml of deionized (DI) water and then mixed with 2 ml of DOX-TPP (4 mg) aqueous solution (Macklin) for 1 min. One milliliter of 2-methylimidazole aqueous solution (HmIm; 64.8 mg/ml; Sigma-Aldrich) was dropwise added to the mixture upon magnetic stirring [1300 revolutions per minute (rpm)] and reacted for 2 hours. The resulting ZIF-67@DOX-TPP nanorobots were collected by centrifugation (13,800*g*, 10 min) and washed twice with DI water. The counterparts, ZIF-67@DOX and ZIF-67 NPs, were prepared by changing the DOX-TPP solution to DOX or omitting the drug solution, respectively, in the aforementioned method.

### Characterization of ZIF-67@DOX-TPP nanorobots

The morphology and elements of fabricated ZIF-67@DOX-TPP nanorobots and ZIF-67 NPs were examined by TEM using a FEI Talos F200X instrument. The hydrodynamic size and zeta potential were measured using Malvern Panalytical Zetasizer Nano ZS90. The XRD analysis was conducted using a D8 ADVANCE diffractometer (Bruker) via Co Kα radiation (λ = 0.179026 nm). UV-vis absorbance spectra were assessed by a Spark multimode microplate reader (Tecan). XPS was tested using ESCALAB 250Xi (Thermo Fisher Scientific). The fluorescent images of ZIF-67@DOX-TPP were captured using CLSM (Zeiss LSM800).

### Drug loading and release

During the fabrication process, various DOX-TPP or DOX inputs (0.5, 1, 2, and 4 mg/ml) were applied upon 2-hour incubation to assess the influence of drug inputs on the loading capacity of ZIF-67–based NPs at room temperature. The parameter of reaction time was evaluated with different durations (0.5, 1, 2, and 4 hours) upon the drug input of 1 mg/ml. The DOX amount was determined by the UV absorbance at 485 nm in accordance with the calibration curve of DOX concentrations.

The drug loading amount was calculated by Eq. 1

$$\text{Loading amount}\;(\mu g/\mathrm{mg})=\frac{\text{Mass of loaded drug}}{\text{Mass of drug-loaded particles}}$$ (1)

The loading efficiency was calculated by Eq. 2

$$\text{Loading efficiency}\;(\%)=\frac{\text{Mass of loaded drug}}{\text{Drug input}}\times 100\%$$ (2)

To evaluate the drug release behavior, 1 mg of ZIF-67@DOX-TPP nanorobots or ZIF-67@DOX NPs were suspended in 2 ml of PBS with different pH values (5.5, 6.5, and 7.4) or various H_2_O_2_ concentrations (0, 50, and 100 μM) and incubated for various durations (1, 2, 3, 4, 8, 12, 16, 20, 24, and 36 hours). The supernatants were collected by centrifugation for UV absorbance detection at 485 nm. The nanorobots after incubation for 12, 24, and 48 hours under each condition were collected for TEM characterization (FEI Talos F200X) to examine their degradations.

### Motion study

ZIF-67@DOX-TPP nanorobots were directly mixed with PBS or 1640 cell culture medium upon various concentrations of H_2_O_2_ (0, 0.1, 1, and 10 mM) on glass substrate, which was covered by a coverslip to prevent drift. The motion videos were recorded by an inverted optical microscope (Eclipse Ti-U, Nikon Instruments Inc.) with a 40× objective using the dark-field condenser. The recorded movies were analyzed using NIS-Elements Advanced Research 5.21.02 software. The diffusion coefficient (*D*_eff_) was measured by fitting MSD data to the equation, MSD (Δ*t*) = 4 *D*_eff_Δ*t*, where Δ*t* represents the time interval. The motion analysis of ZIF-67 NPs was also conducted in 0.1 mM H_2_O_2_.

Regarding the motion in cytoplasm extract, T24 bladder tumor cells (1 × 10^7^ cells) were suspended in PBS solution (0.2 ml) and treated with three freeze-thaw cycles to rupture cell membranes (*68*, *69*). Cytoplasm solution was obtained by centrifuging the mixture and collecting the supernatant. Then, nanorobots were added to extracted cytoplasm solution with various H_2_O_2_ concentrations for motion analysis.

### Cytotoxicity of ZIF-67 NPs

The cell lines used in this study were all obtained from the American Type Culture Collection. T24, BEND3, and SV-HUC-1 cells were seeded in a 96-well plate (1 × 10^4^ cells per well) and incubated overnight. Then, the suspension was removed, followed by adding ZIF-67 NPs with various concentrations (8, 16, 32, 64, and 128 μg/ml) and incubating for 24 and 48 hours. The cell viability was examined by Cell Counting Kit-8 (CCK-8) assay (Meilunbio). The absorbance at 450 nm was measured using a Spark multimode microplate reader (Tecan).

### Lysosomal escape of ZIF-67@DOX-TPP nanorobots

T24 cells (2 × 10^5^ cells) were first cultured in a cell culture dish (35 mm; NEST) overnight. After removing the cell culture media, cells were incubated with ZIF-67@DOX-TPP nanorobots with a DOX concentration of 1 μg/ml for 1 and 8 hours. The cellular lysosome and nucleus were sequentially labeled with LysoTracker Green (50 nM, excitation/emission = 504/511 nm; Meilunbio) and Hoechst 33342 (1 μM, excitation/emission = 361/497 nm) for 30 min each. The cells were washed with PBS three times to remove free dyes, followed by fluorescent imaging using the CLSM (Zeiss LSM800). Five typical fluorescence images of DOX and LysoTracker Green channels were used to calculate the Pearson's correlation coefficient via ImageJ software.

### Synthesis of ZIF-8 NPs and DOX-loaded counterparts

To fabricate ZIF-8 NPs, 4 ml of HmIm (30.5 mg/ml) aqueous solution was dropwise added into 1 ml of (CH_3_COO)_2_Zn·2H_2_O (ZnAc_2_·2H_2_O; 50 mg/ml; Aladdin) under magnetic stirring (1300 rpm) and reacted for 3 hours. The resulting ZIF-8 NPs were collected by centrifugation (13,800*g*, 10 min) and washed twice with DI water.

To prepare drug-loaded ZIF-8 NPs, DOX or DOX-TPP was first mixed with ZnAc_2_·2H_2_O and then reacted with HmIm solution to obtain ZIF-8@DOX or ZIF-8@DOX-TPP, respectively. The morphology of ZIF-8 and ZIF-8@DOX-TPP NPs were characterized using TEM (FEI Talos F200X). The motion performance of ZIF-8@DOX-TPP NPs was examined in PBS solution with various concentrations of H_2_O_2_ (0, 0.1, 1, and 10 mM). The movement of ZIF-8 NPs was explored in 0.1 mM H_2_O_2_.

### Intracellular movement analysis

T24 cells, 4T1 cells, and SV-HUC-1 cells were cultured in cell culture dishes (2 × 10^5^ cells per dish) overnight. Then, ZIF-67@DOX-TPP nanorobots (1 μg/ml of DOX) were added for 6-hour incubation. The cells were washed three times with PBS to remove free nanorobots. The intracellular movement of the nanorobots was recorded using the CLSM with a 63× oil objective (~3.15 frames per second). The motion videos were analyzed using ImageJ software. The motion behavior observation of ZIF-8@DOX-TPP NPs was also conducted in PBS solution with various H_2_O_2_ concentrations and T24 cancer cells, acting as the counterpart without H_2_O_2_ catalysis.

### Mitochondrial targeting behavior

T24 cells or 4T1 cells were seeded in cell culture dishes (2 × 10^5^ cells per dish) and cultured overnight. The cells were cocultured with DOX, DOX-TPP, ZIF-8@DOX, ZIF-8@DOX-TPP, ZIF-67@DOX, and ZIF-67@DOX-TPP nanorobots for 12 hours. The DOX concentration in drug-loaded groups was the same as 1 μg/ml. After three-time washing with PBS, the cells were incubated with MitoTracker Green (100 nM, excitation/emission = 490/516 nm; Meilunbio) and Hoechst 33342 (1 μM) for 30 min to label mitochondria and nuclei, respectively. The fluorescent images of cells were captured using the CLSM. Five typical fluorescence images of DOX and MitoTracker Green channels were used to calculate the Pearson's correlation coefficient via ImageJ software. The nanorobots were also incubated with T24 cells for various durations (2, 4, 6, and 12 hours) to evaluate the mitochondrial targeting effect of nanorobots at different time points.

### In vitro anticancer activity

To assess the early mitochondrial apoptosis, T24 cells (2 × 10^5^ cells) were seeded in cell culture dishes and incubated overnight. Then, cells were cultured with various solutions, including PBS, ZIF-8, ZIF-67, ZIF-8@DOX, ZIF-67@DOX, ZIF-8@DOX-TPP, and ZIF-67@DOX-TPP. The incubation time was set to 8 hours to avoid excessive damage to the mitochondria, which may lead to inaccurate evaluation of early apoptosis. The DOX concentrations in the drug groups were equivalent to 5 μg/ml. After three-time washing with PBS, the cells were labeled with JC-1 dye (10 μg/ml; J-aggregate, excitation/emission = 585/590 nm and J-monomer, excitation/emission = 514/529 nm) for 20 min. The fluorescence images were captured using the CLSM. The fluorescence ratio of aggregate and monomer dyes was quantified via ImageJ software.

To explore the in vitro anticancer efficacy, T24, 4T1, and BIU-87/ADR cells were separately seeded in the 96-well plate with a density of 1 × 10^4^ cells per well and cultured overnight. Then, cells were incubated in various solutions, including PBS, DOX-TPP, ZIF-8@DOX-TPP, ZIF-67@DOX, and ZIF-67@DOX-TPP nanorobots. After 48-hour incubation, the cell viability was evaluated using CCK-8 assay.

To explore the impact of H_2_O_2_ decomposition and oxygen generation on the anticancer efficacy of DOX-TPP, the mixture of free ZIF-67 NPs and H_2_O_2_ was used to generate oxygen. T24 cells (1 × 10^4^ cells per well) were seeded in the 96-well plate and cultured overnight. Then, cells were incubated with various PBS solutions containing DOX-TPP, DOX-TPP and H_2_O_2_ (DOX-TPP + H_2_O_2_), DOX-TPP and ZIF-67 NPs (DOX-TPP + ZIF-67), or a mixture of DOX-TPP, H_2_O_2_, and ZIF-67 (DOX-TPP + H_2_O_2_ + ZIF-67). Two DOX-TPP concentrations, 5 and 10 μg/ml were used. The concentrations of ZIF-67 NPs and H_2_O_2_ were fixed at 20 μg/ml and 20 μM, respectively. After 48-hour incubation, the cell viability was evaluated using CCK-8 assay.

Western blot was conducted to verify the mitochondria-mediated cell apoptosis. T24 cells were incubated with PBS, DOX-TPP, ZIF-8@DOX-TPP, ZIF-67@DOX, and ZIF-67@DOX-TPP for 36 hours, where the DOX concentration of drug-loaded groups was consistent at 5 μg/ml. Then, the cells were harvested and lysed using a cell lysis buffer (Meilunbio). The protein concentration of each group was determined by a bicinchoninic acid assay. Equivalent proteins of each group were separated by SDS–polyacrylamide gel electrophoresis and transferred to a polyvinylidene fluoride membrane under a standard procedure. Then, the membranes were cultured with primary antibodies (ProteinTech and Huaan Biotechnology) overnight, followed by incubation with secondary antibody for 1 hour to tag the target proteins.

### In vitro evaluation of cancer metastasis

The migration capability of T24 cells and 4T1 cells was evaluated by scratch wound healing assay. T24 cells or 4T1 cells were seeded in a 12-well plate and cultured until 80 to 90% confluence. A sterile pipette tip (200 μl) was used to scratch a straight line through the cell monolayer, acting as the wound. Then, cells were incubated with various solutions, including PBS, ZIF-8@DOX-TPP, ZIF-67@DOX, and ZIF-67@DOX-TPP, for 12 hours. The DOX dosage in drug groups was the same as 2.5 μg/ml. The scratched wound was captured using an optical microscope at the beginning (0 hours) and after 12-hour incubation. The scratch area was calculated by ImageJ software to quantify wound healing rates.

A transwell assay was performed to evaluate the invasion competence of T24 cells and 4T1 cells. First, 80 μl of Matrigel (1:8 dilution; Corning) was added into the upper chamber of the transwell (24-well, 8.0-μm pore size) and cultured at 37°C for 3 hours. Next, 100 μl of serum-free 1640 medium containing T24 cells (5 × 10^4^ cells) was injected into the upper chamber. A total of 600 μl of 1640 medium with 10% fetal bovine serum was added into the lower chamber. Afterward, various solutions including PBS, ZIF-8@DOX-TPP, ZIF-67@DOX, and ZIF-67@DOX-TPP (equivalent DOX dose of 2.5 μg/ml) were added to the upper layer of the transwell and incubated for 24 hours. Then, cells in the upper chamber were removed and washed with PBS three times. The invaded cells that crossed the Matrigel and membrane to the lower surface were fixed by methanol for 20 min. Last, the cells were stained with 0.1% crystal violet for 10 min for fluorescent imaging. The invasion cells were quantified by counting five representative images via ImageJ software.

### Animal care

All mice in the present study were purchased from GemPharmatech Co. Ltd. The care and use of animals were in strict accordance with the guidelines of the Animal Use and Care Administrative Advisory Committee of Shenzhen Luohu Hospital Group.

### In vivo antitumor evaluation

Tumor-bearing mice were established by subcutaneously injecting 5 × 10^6^ of T24 cells into BALB/c nude mice (6-week-old males). When the tumor volume reached approximately 100 mm^3^, the mice were randomly divided into seven groups (*n* = 5) and were intratumorally injected with PBS, ZIF-67, DOX-TPP, ZIF-8@DOX-TPP, ZIF-67@DOX, and ZIF-67@DOX-TPP nanorobots, respectively. The DOX dosages for the mice in drug groups were equivalent to 2.5 mg/kg. The DOX concentration of the injection solution was unified to 2.5 mg/ml. Depending on the body weight of the mice, a total volume of approximately 27 μl on average was intratumorally injected. Injection volumes from 10 to 50 μl have been verified to meet the operation standard and safety requirement for intratumoral administration (*70*–*72*). Such administration was repeated every 2 days for a total of six times. The tumor volume and weight of tumor-bearing mice were monitored during the treatment course. The mice were euthanized at the end point of treatment (day 13). The tumors were resected for histological staining, including H&E, TUNEL, and Ki67, to examine the anticancer effect. Main organs (heart, liver, spleen, lungs, and kidneys) were also dissected for H&E staining to assess the biosafety of the nanorobots. The liver slices from the control (PBS) and nanorobot groups and the tumor slice from the nanorobot group were immunohistochemically stained using HLA-DRA polyclonal antibody (ABclonal, A11787) to evaluate the possible liver metastasis of subcutaneous T24 tumor.

The unilateral 4T1 orthotopic breast tumor model was established by injecting 5 × 10^5^ of 4T1 cells into the breast fat pad of female BALB/c mice (6 weeks old). When the tumor volume reached approximately 100 mm^3^, the mice were randomly divided into six groups (*n* = 5) and were intratumorally administrated with PBS, ZIF-67, DOX-TPP, ZIF-8@DOX-TPP, ZIF-67@DOX, and ZIF-67@DOX-TPP nanorobots, respectively. DOX dosages for the mice in drug groups were standardized to 2.5 mg/kg. The DOX concentration in the injection solution was unified to 2.5 mg/ml. The injection volume was adjusted to around 22 μl on average based on the detailed mouse body weight. The administration was repeated every 3 days for a total of six times. The tumor volume and body weight of each mouse was recorded throughout the treatment course. The mice were euthanized at the end point (day 22). The primary tumors were harvested to measure the weights and photographed. The lungs were resected and fixed in Bouin's solution (Phygene). The metastatic nodules on the surface of the lungs were stained light yellow for facile counting. Last, H&E staining of these lung tissues was conducted.

### Statistical analysis

Quantitative data are expressed as means ± SD or means ± SEM when appropriate, as shown in figure captions. Comparison of multiple groups was performed using one-way analysis of variance (ANOVA) with a Tukey post hoc test. Statistical significance is represented as *P < 0.05; **P < 0.01; ***P < 0.001; ****P < 0.0001.

## Notes

View/request a protocol for this paper from *Bio-protocol*.
