# Supplementary material for: Autonomous metal-organic framework nanorobots for active mitochondria-targeted cancer therapy
Source: Sci Adv. 2023 Jun 9;9(23):eadh1736. doi: 10.1126/sciadv.adh1736 (PMC10256165; doi:10.1126/sciadv.adh1736)
Supplement: Supplementary file 1 — Figs. S1 to S20 Legends for movies S1 to S7 [file sciadv.adh1736_sm.pdf]

Science Advances
AAAS

Supplementary Materials for

Autonomous metal-organic framework nanorobots for active mitochondria-targeted cancer therapy

Xiqi Peng *et al.*

Corresponding author: Songsong Tang, sstang@caltech.edu; Wei Gao, weigao@caltech.edu;
Song Wu, wusong@szu.edu.cn

Sci. Adv. **9**, eadh1736 (2023)
DOI: 10.1126/sciadv.adh1736

The PDF file includes:

Figs. S1 to S20
Legends for movies S1 to S7

Other Supplementary Material for this manuscript includes the following:

Movies S1 to S7

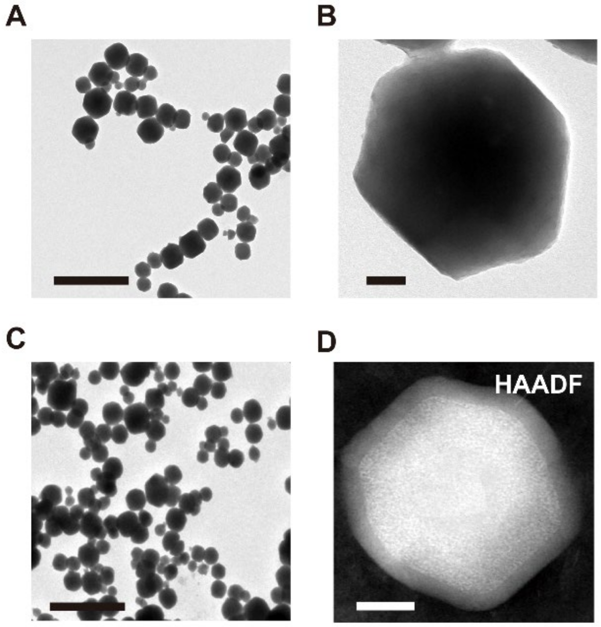

Fig. S1. TEM images of ZIF-67 NPs (A-B) and ZIF-67@DOX-TPP nanorobots (C-D). Scale bars: 1 μm (**A,C**); 50 nm (**B,D**).

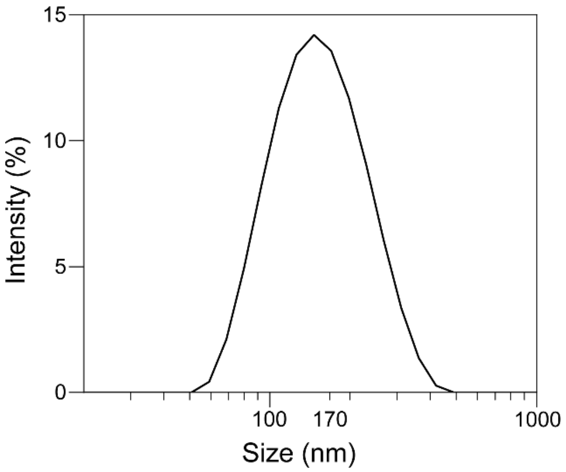

Fig. S2. Hydrodynamic size distribution of ZIF-67@DOX-TPP nanorobots.

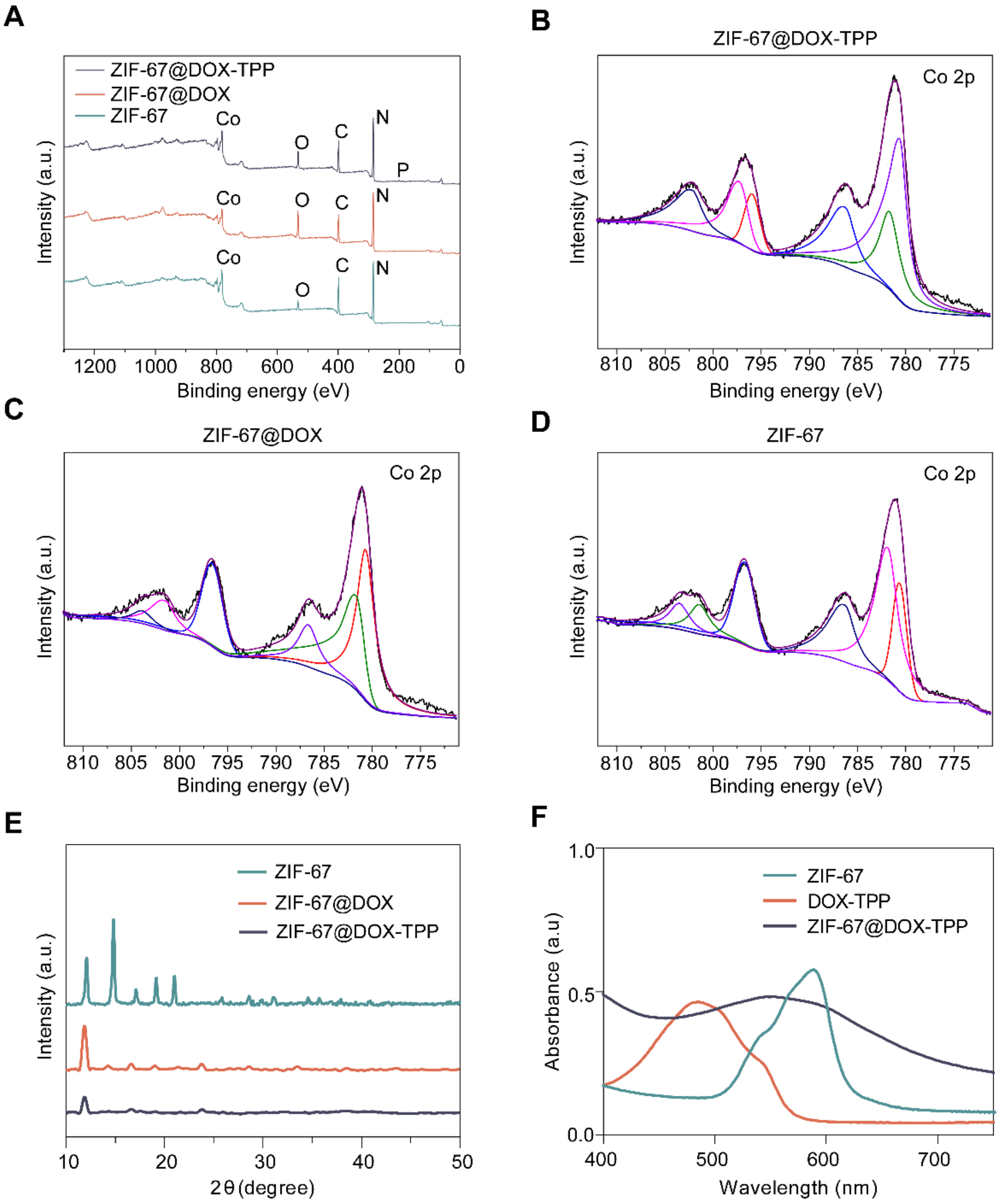

Fig. S3. Characterization of ZIF-67@DOX-TPP nanorobots and their counterparts. (**A**) Wide scan of XPS spectra. Co 2p spectra of (**B**) ZIF-67@DOX-TPP nanorobots, (**C**) ZIF-67@DOX, and (**D**) ZIF-67 NPs. (**E**) XRD spectra and (**F**) UV-vis absorption of ZIF-67 NPs, ZIF-67@DOX, and ZIF-67@DOX-TPP nanorobots.

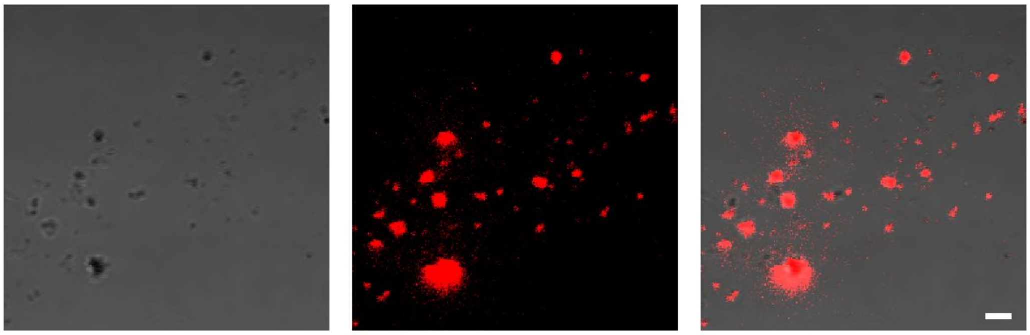

Fig. S4. Fluorescent images of ZIF-67@DOX-TPP nanorobots. Scale bar, 2 μm.

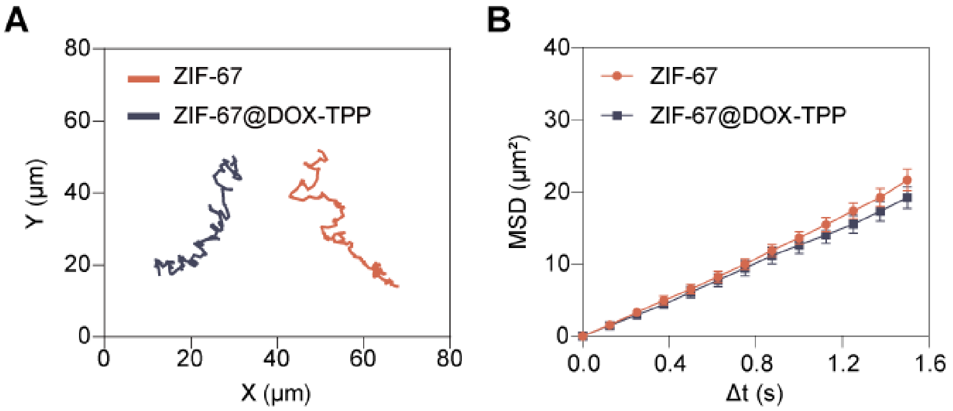

Fig. S5. Propulsion analysis of ZIF-67 NPs and ZIF-67@DOX-TPP nanorobots in PBS solution with 100 μM H_2O_2. (**A**) Typical motion trajectories (over 20 s). (**B**) mean square displacement (MSD) (n=15; mean ± SEM).

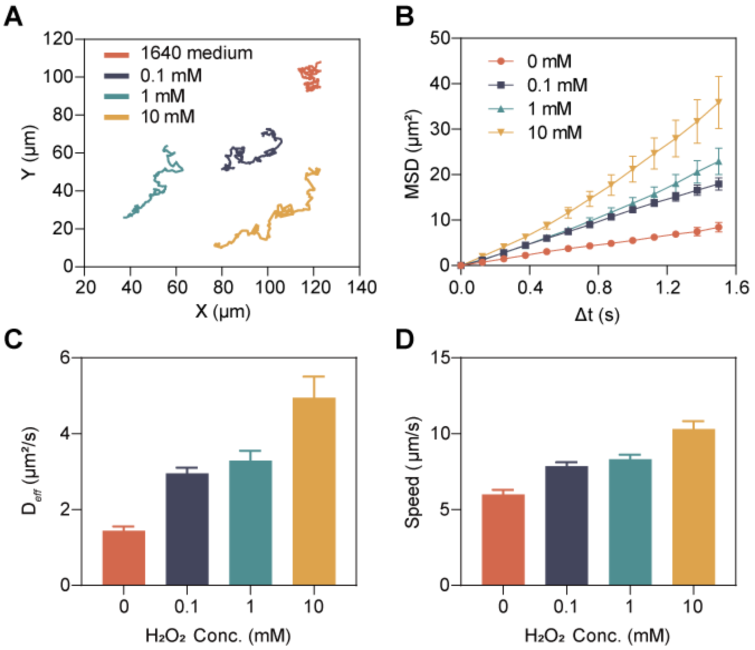

Fig. S6. Propulsion analysis of ZIF-67@DOX-TPP nanorobots in 1640 culture medium with various H_2O_2 concentrations. (**A**) Typical motion trajectories (over 20 s). (**B**) MSD. (**C**) D_{eff}. (**D**) Speed (n = 15; mean ± SEM).

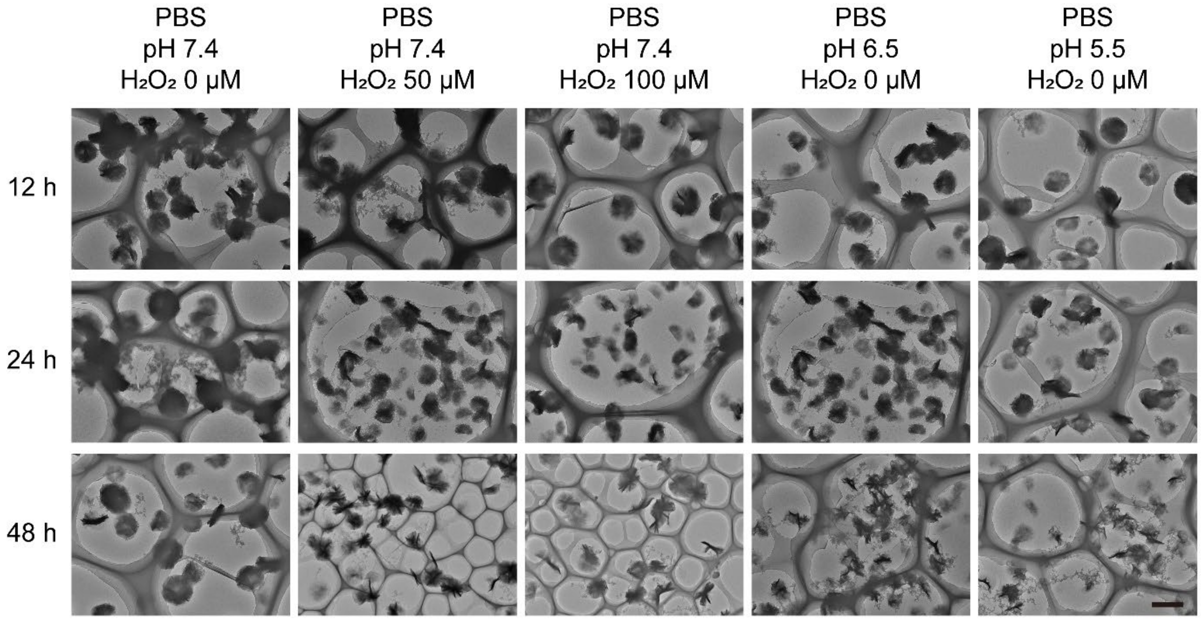

Fig. S7. TEM images of ZIF-67@DOX-TPP nanorobots after incubation in PBS solution with different pH and H_2O_2 concentrations for 12, 24 and 48 hours. Scale bar, 200 nm.

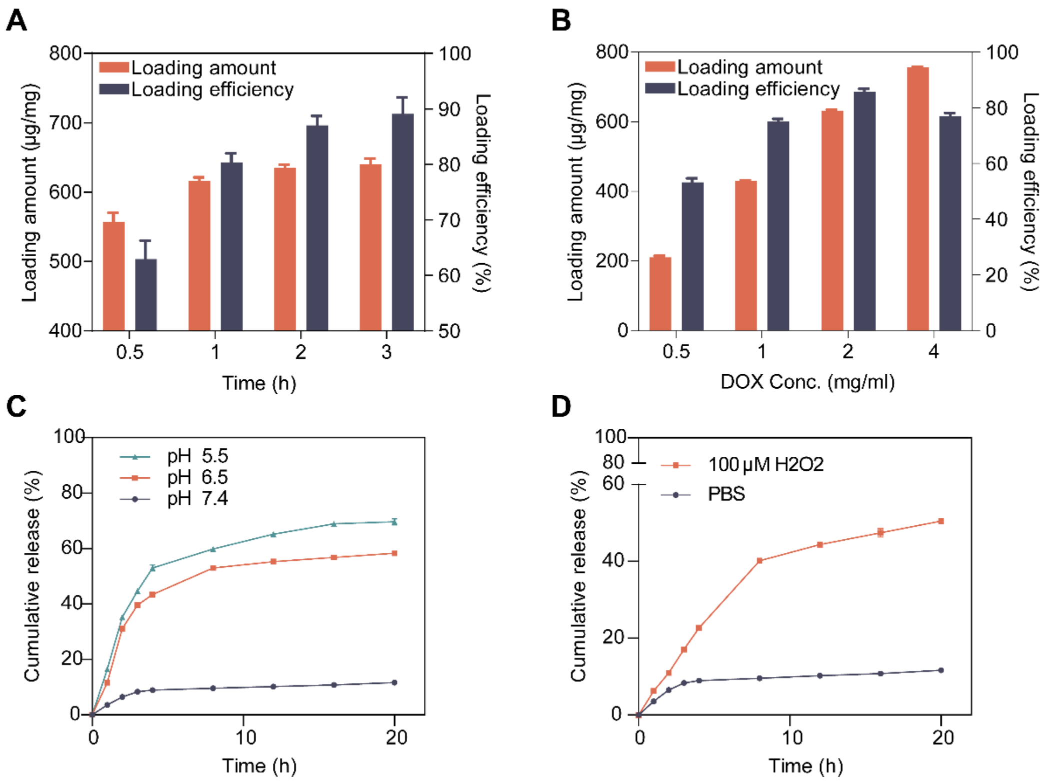

Fig. S8. Drug loading and release profiles of ZIF-67@DOX NPs. Drug loading capacity of ZIF-67@DOX NPs upon various incubation time (**A**) and DOX input concentrations (**B**) (n = 3; mean ± SD). The cumulative release of DOX from NPs upon different pH values (**C**) and H_2O_2 concentrations (**D**) (n = 3; mean ± SD).

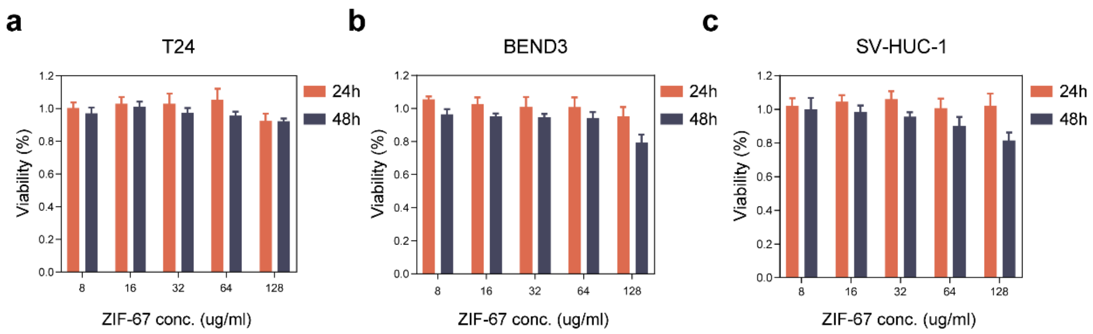

Fig. S9. Cytotoxicity of ZIF-67 NPs to different cell lines. (**A**) T24 bladder cancer cells. (**B**) BEND3 brain endothelial cells. (**C**) SV-HUC-1 urothelial cells (n = 3; mean ± SD).

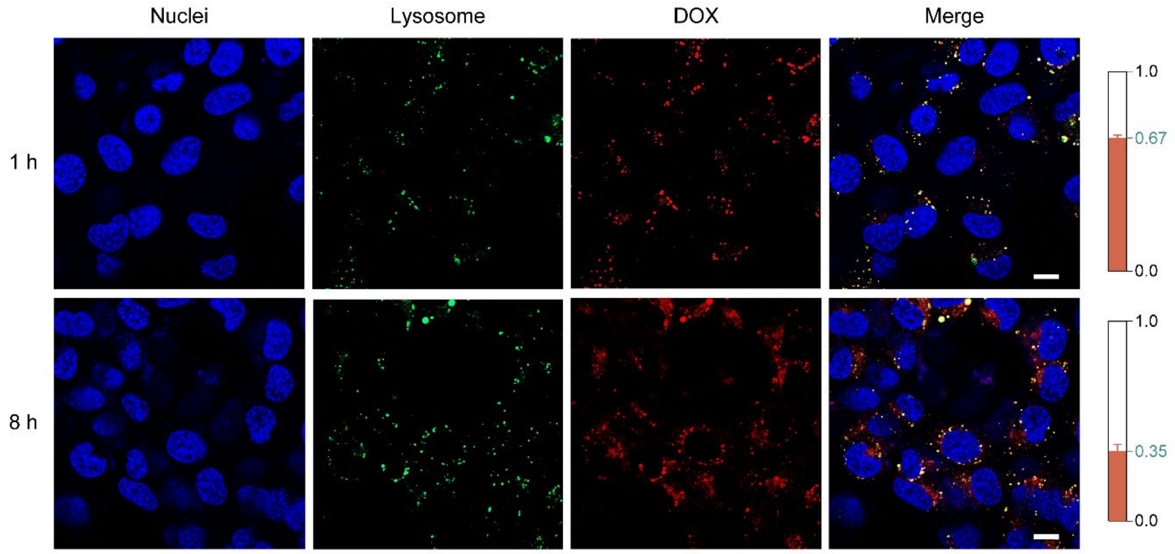

Fig. S10. The co-localization of ZIF-67@DOX-TPP nanorobots and lysosomes in T24 bladder cancer cells after short and long incubation durations. The nuclei and lysosome were stained with Hoechst 33342 (blue) and LysoTracker Green dye, respectively. The right columns show the calculated PCC of the colocalization of lysosome and nanorobots in T24 cells (n = 5; mean ± SD). Scale bars, 10 μm.

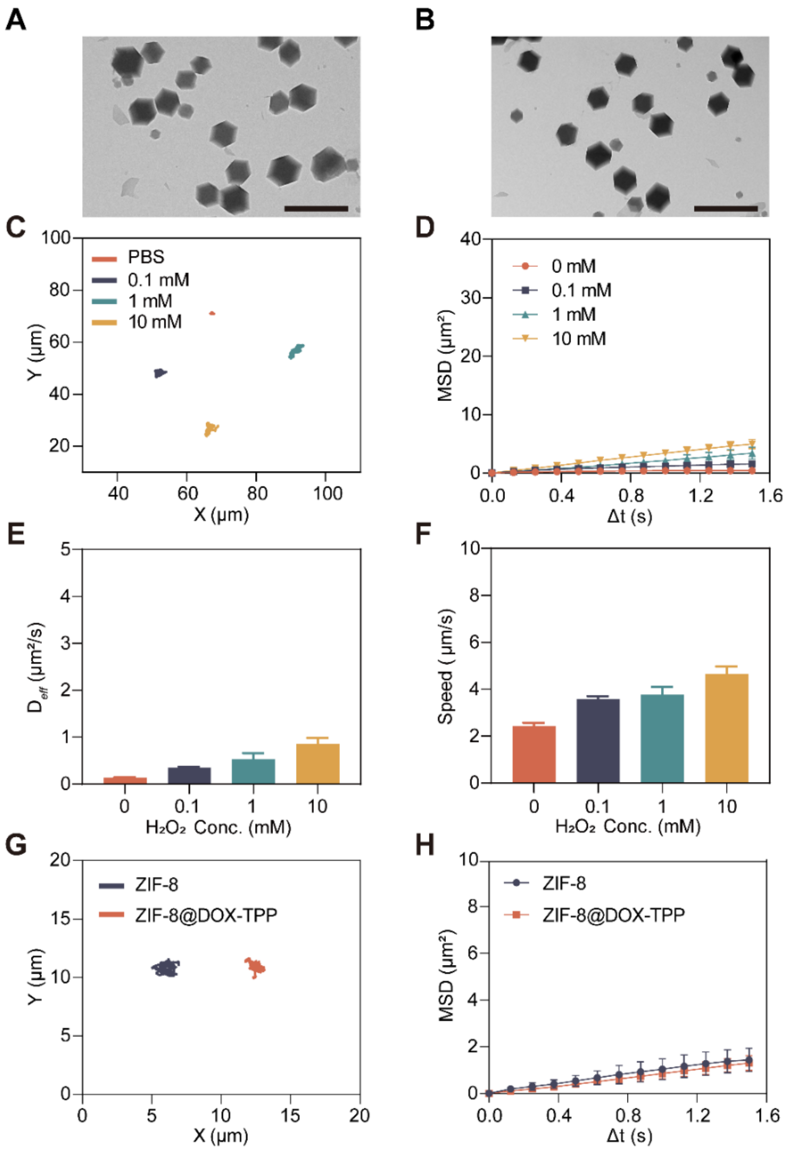

Fig. S11. Morphology characterization and propulsion analysis of ZIF-8 and ZIF-8@DOX-TPP NPs. TEM images of ZIF-8 NPs (**A**) and ZIF-8@DOX-TPP NPs (**B**). Scale bars, 500 nm. Typical motion trajectories (over 20 s) (**C**), MSD (**D**), Deff (**E**), and speed (**F**) of ZIF-8@DOX-TPP NPs in PBS with various H_2O_2 concentrations. Comparison of typical motion trajectories (over 20 s) (**G**) and MSD (**H**) of ZIF-8 and ZIF-8@DOX-TPP NPs in PBS with 100 μM H_2O_2. (n = 15; mean ± SEM).

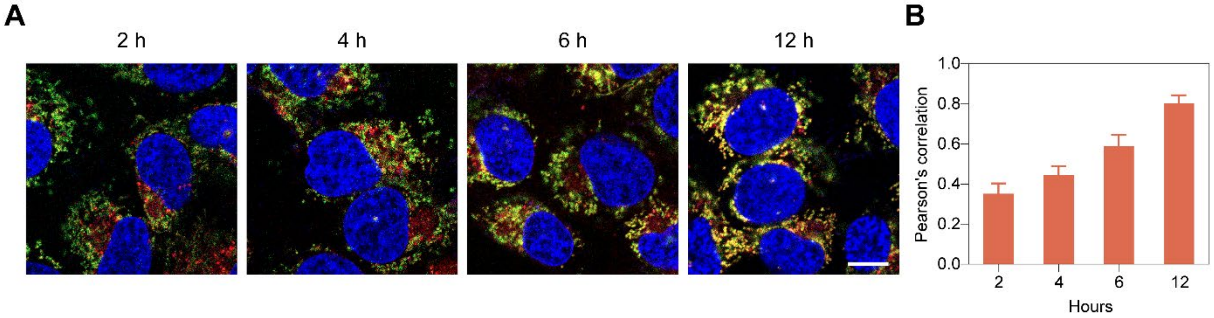

Fig. S12. Representative fluorescence images showing mitochondrial colocalization in T24 cells upon incubation with ZIF-67@DOX-TPP nanorobots for 2, 4, 6, 12 hours and corresponding PCC value (n = 5; mean ± SD). Scale bar, 10 μm.

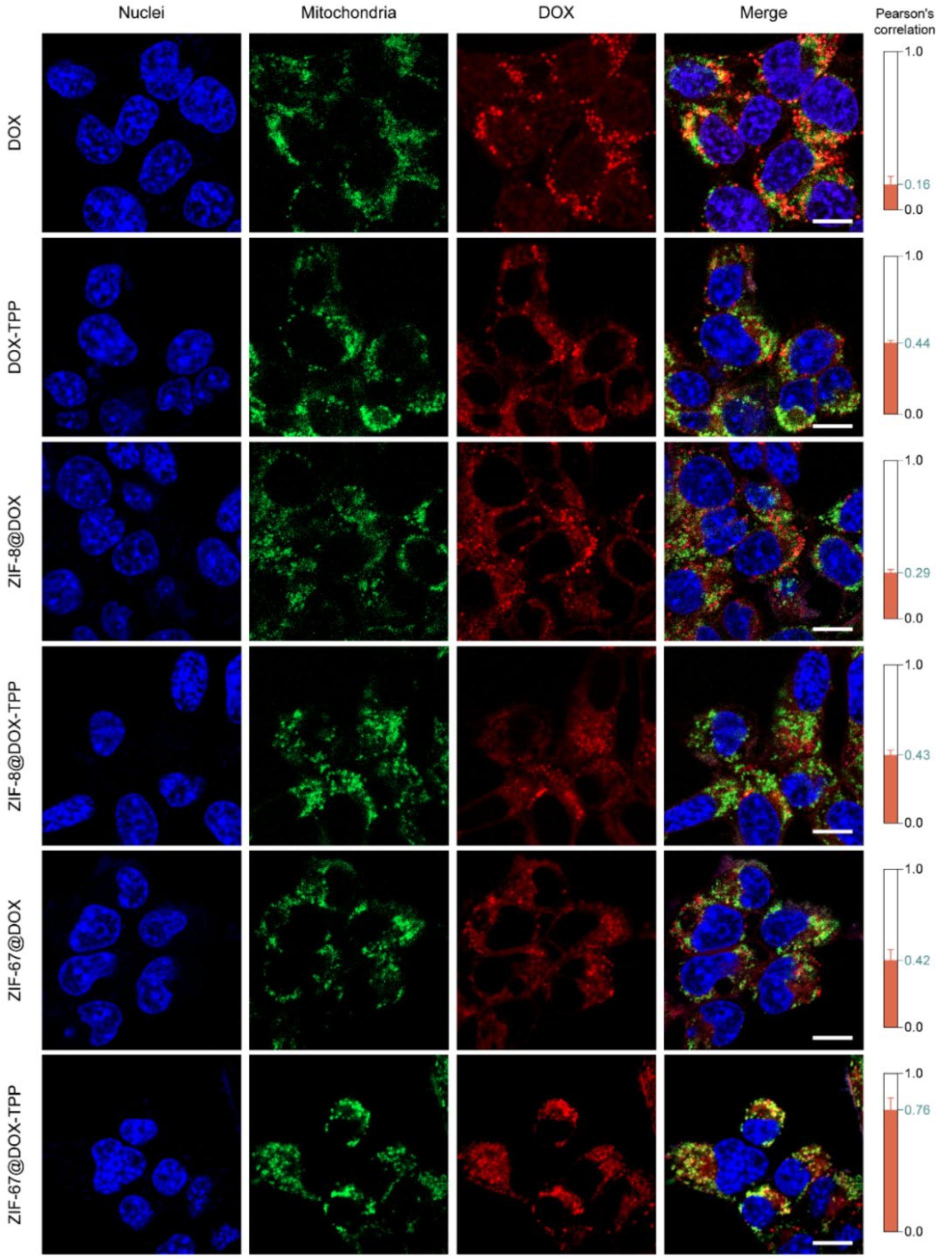

Fig. S13. Representative fluorescence images showing mitochondrial colocalization in 4T1 cells upon various incubations for 12 hours, including DOX, DOX-TPP, ZIF-8@DOX, ZIF-8@DOX-TPP, ZIF-67@DOX, and ZIF-67@DOX-TPP nanorobots. (n=5, mean ± SD). Scale bars, 10 μm.

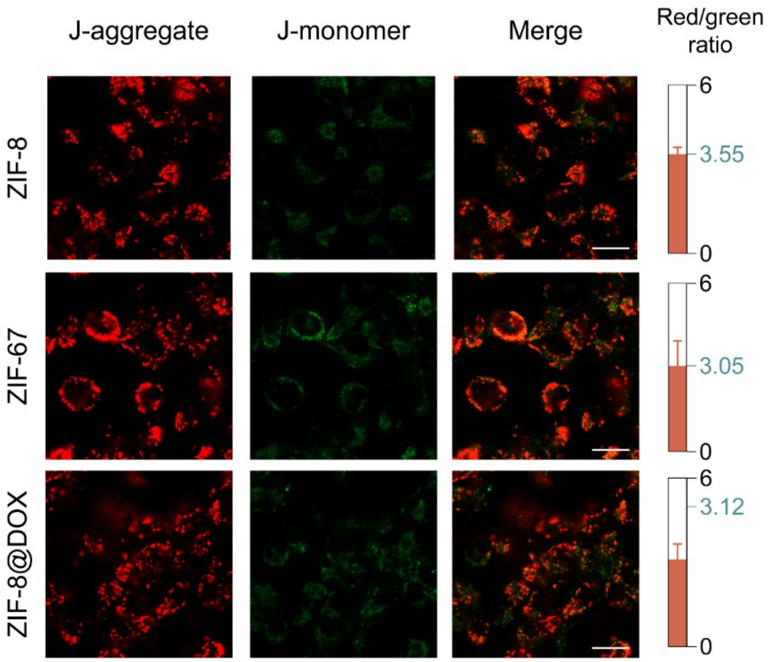

Fig. S14. JC-1 staining images and calculated J-aggregate/J-monomer ratios of T24 bladder cancer cells that incubated upon various conditions for 8 hours (n = 5; mean ± SD)**.** Scale bars, 20 μm.

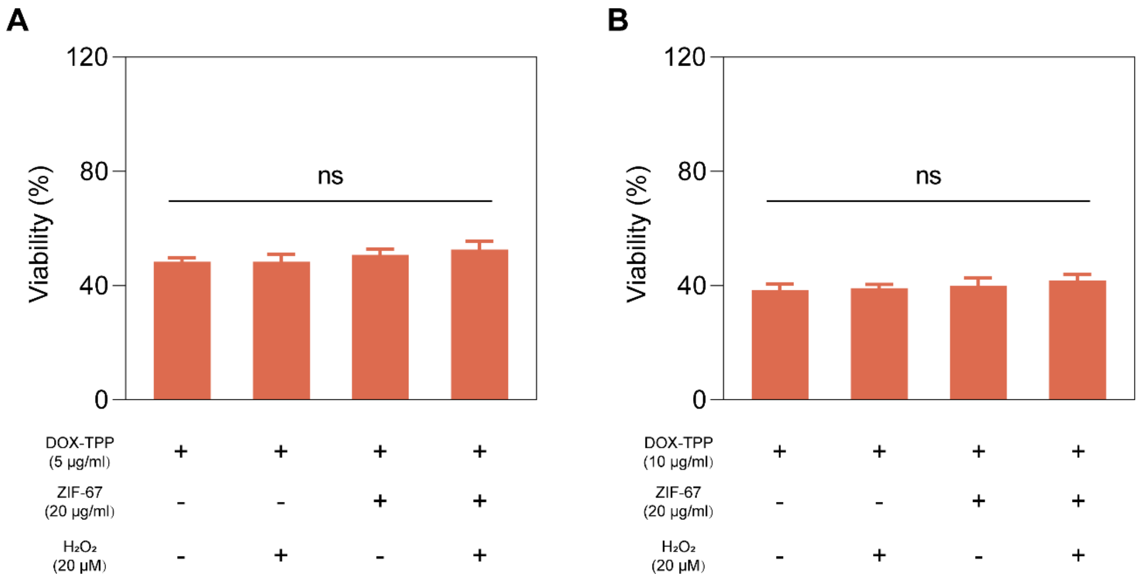

Fig. S15. The effect of H_2O_2 decomposition on the anticancer efficacy of DOX-TPP after incubation with T24 cells for 48 hours (n=3, mean ± SD).

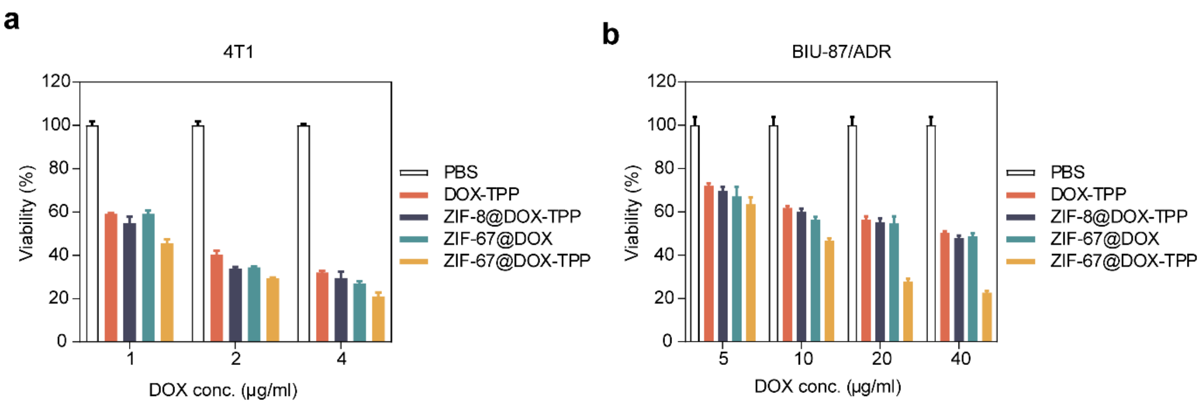

Fig. S16. The viability of 4T1 breast cancer cells (A) and DOX-resistant BIU-87/ADR bladder cancer cells (B) after incubation with various treatments for 48 hours (n = 3; mean ± SD).

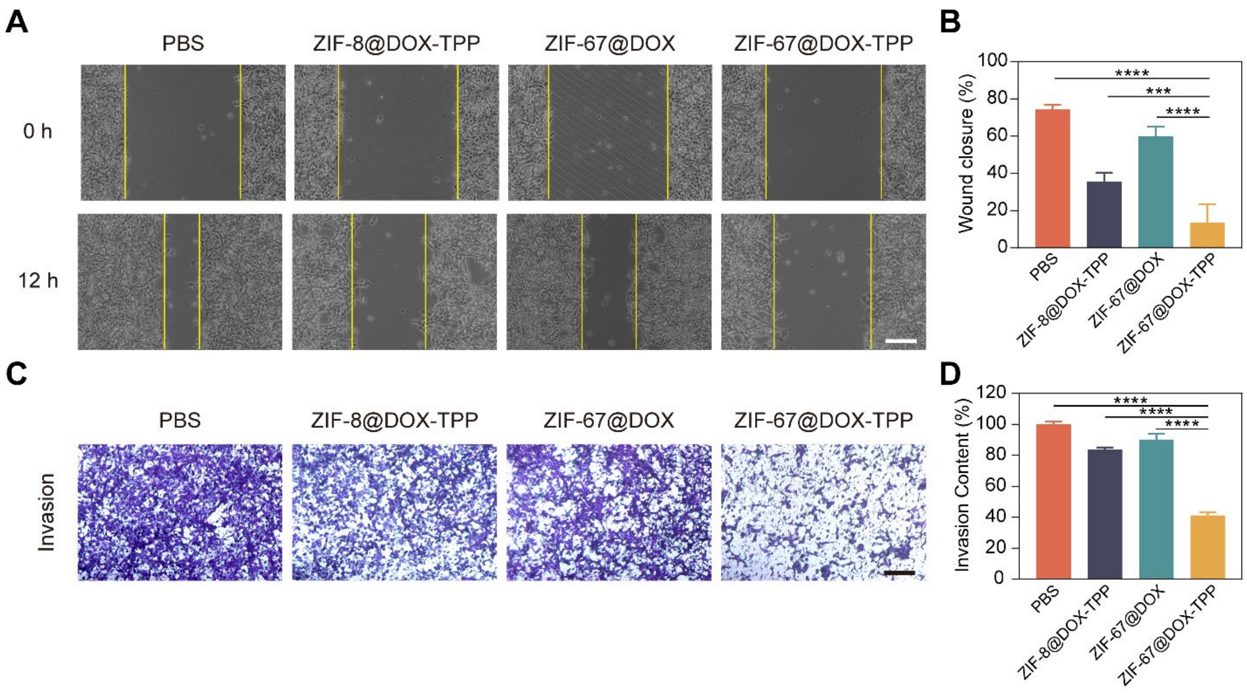

Fig. S17. In vitro evaluation of ZIF-67@DOX-TPP nanorobots for metastasis inhibition in 4T1 breast cancer cells. (A) Optical images showing in vitro wound healing assay and (B) corresponding wound closure percentages. The 'wound' (cell gap) was built by a straight scratch across 4T1 cancer cells (0 h). The wound closure rate was examined after incubation with nanorobots and other control groups for 12 hours. (n = 5; mean ± SD). Scale bar, 100 μm. (C) Images of invaded 4T1 cells across the Matrigel barrier after treatment with nanorobots and other control groups in the upper chamber of transwell assay and (G) corresponding invasive contents (n = 5; mean ± SD). Scale bar, 200 μm. ***$P < 0.001$, ****$P < 0.0001$; One-way ANOVA.

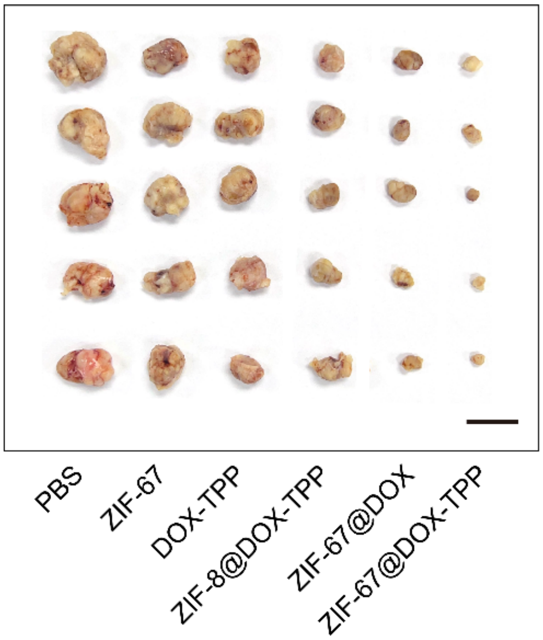

Fig. S18. Images of resected subcutaneous T24 tumors at the endpoint of the treatment course with nanorobots and other constructs. Scale bar, 2 cm.

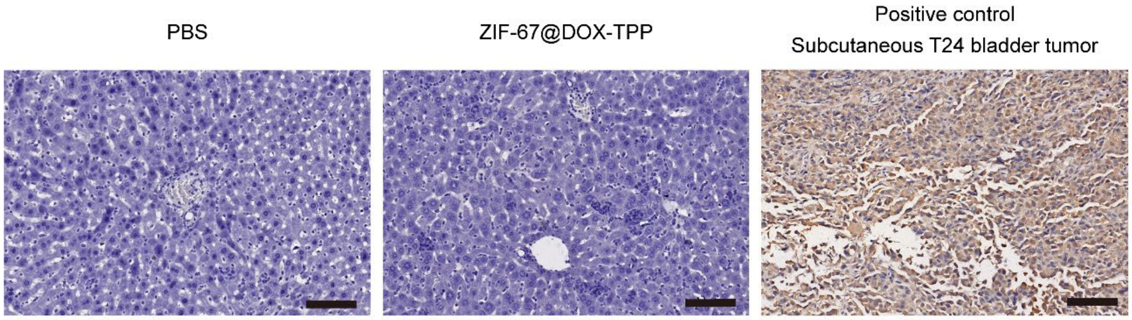

Fig. S19. Immunohistochemical staining of resected liver slices from the group of PBS (A) and ZIF-67@DOX-TPP nanorobot (B) and T24 tumor slice of the nanorobot group (C) after the treatment course of subcutaneous tumor model. HLA-DRA pAb was used to label the expressed HLA-DRA on the surface of human-derived T24 cancer cells (Brown). Scale bar, 100 μm.

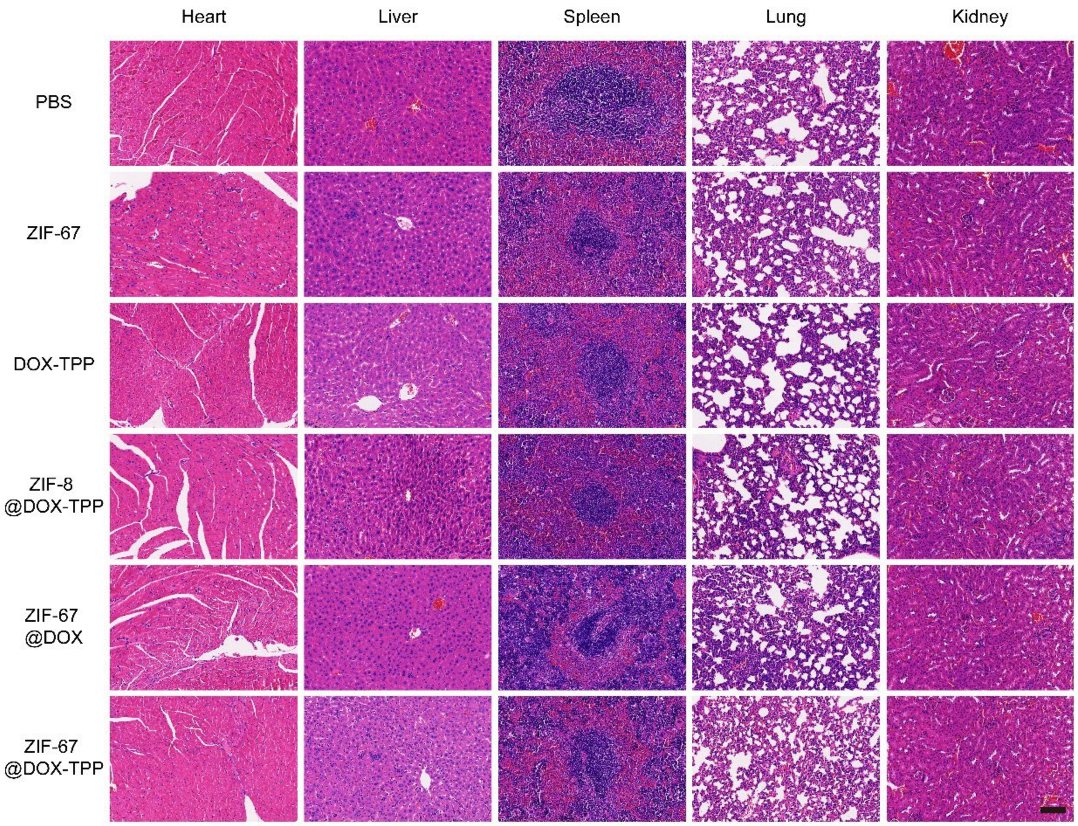

Fig. S20. H＆E staining of histological sections from main organs, including heart, liver, spleen, lung, and kidney, resected at the endpoint of the treatment course from T24 tumor-bearing mice that incubated with ZIF-67@DOX-TPP nanorobots and other control groups. Scale bar, 100 μm.

Movie S1. Propulsion performance of ZIF-67@DOX-TPP nanorobots in PBS solution with different H_2O_2 concentrations.

Movie S2. Motion comparison of ZIF-67 NPs and ZIF-67@DOX-TPP nanorobots in PBS solution with 100 μM H_2O_2.

Movie S3. Propulsion performance of ZIF-67@DOX-TPP nanorobots in 1640 medium with different H_2O_2 concentrations.

Movie S4. Propulsion performance of ZIF-67@DOX-TPP nanorobots in cytoplasm extract with different H_2O_2 concentrations.

Movie S5. Intracellular movement of ZIF-67@DOX-TPP nanorobots and ZIF-8@DOX-TPP NPs in different cells.

Movie S6. Propulsion performance of ZIF-8@DOX-TPP NPs in PBS solution with different H_2O_2 concentrations.

Movie S7. Motion comparison of ZIF-8 NPs and ZIF-8@DOX-TPP NPs in PBS solution with 100 μM H_2O_2.
